# The systematic use of evidence‐based methodologies and technologies enhances shared decision‐making in the 2018 International Consensus Conference on Patient Blood Management

**DOI:** 10.1111/vox.12852

**Published:** 2019-11-10

**Authors:** Hans Van Remoortel, Kari Aranko, Markus M. Mueller, Emmy De Buck, Dana Devine, Gilles Folléa, Patrick Meybohm, Pierre Tiberghien, Erica M. Wood, Philippe Vandekerckhove, Erhard Seifried

**Affiliations:** ^1^ Centre for Evidence‐Based Practice (CEBaP) Belgian Red Cross Mechelen Belgium; ^2^ European Blood Alliance (EBA) Amsterdam The Netherlands; ^3^ Finnish Red Cross Blood Service Helsinki Finland; ^4^ German Red Cross Blood Transfusion Service Frankfurt/Main Germany; ^5^ Department of Public Health and Primary Care Faculty of Medicine KU Leuven Leuven Belgium; ^6^ Canadian Blood Services Ottawa ON Canada; ^7^ Société Française de Transfusion Sanguine (SFTS) Paris France; ^8^ Department of Anaesthesiology Intensive Care Medicine and Pain Therapy University Hospital Frankfurt Frankfurt/Main Germany; ^9^ Etablissement Français du Sang (EFS) Saint‐Denis France; ^10^ International Society of Blood Transfusion (ISBT) Amsterdam The Netherlands; ^11^ Transfusion Research Unit Department of Epidemiology and Preventive Medicine Monash University Melbourne Vic Australia; ^12^ Belgian Red Cross Mechelen Belgium

**Keywords:** anemia, patient blood management, red cell components, transfusion

## Abstract

**Background and objectives:**

Patient Blood Management (PBM) aims to optimize the care of patients who might need a blood transfusion. The International Consensus Conference on PBM (ICC‐PBM) aimed to develop evidence‐based recommendations on three topics: preoperative anaemia, red blood cell transfusion thresholds and implementation of PBM programmes. This paper reports how evidence‐based methodologies and technologies were used to enhance shared decision‐making in formulating recommendations during the ICC‐PBM.

**Materials & Methods:**

Systematic reviews on 17 PICO (Population, Intervention, Comparison, Outcomes) questions were conducted by a Scientific Committee (22 international topic experts and one methodologist) according to GRADE (Grades of Recommendation, Assessment, Development and Evaluation) methodology. Evidence‐based recommendations were formulated using Consensus Development Conference methodology.

**Results:**

We screened 17 607 references and included 145 studies. The overall certainty in the evidence of effect estimates was generally low or very low. During the ICC, plenary sessions (100–200 stakeholders from a range of clinical disciplines and community representatives) were followed by closed sessions where multidisciplinary decision‐making panels (>50 experts and patient organizations) formulated recommendations. Two chairs (content‐expert and methodologist) moderated each session and two rapporteurs documented the discussions. The Evidence‐to‐Decision template (GRADEpro software) was used as the central basis in the process of formulating recommendations.

**Conclusion:**

This ICC‐PBM resulted in 10 clinical and 12 research recommendations supported by an international stakeholder group of experts in blood transfusion. Systematic, rigorous and transparent evidence‐based methodology in a formal consensus format should be the new standard to evaluate (cost‐) effectiveness of medical treatments, such as blood transfusion.

## Introduction

Patient Blood Management (PBM) is a patient‐focused, evidence‐based and multidisciplinary approach to optimize both the management of patients and transfusion of blood products for quality and effective patient care. It is designed to improve patient outcomes through the safe and rational use of blood and blood products and by minimizing unnecessary exposure to blood products 1.

Key areas in PBM include diagnosing and treating perioperative anaemia, implementing blood‐saving measures throughout the course of diagnosis and treatment, and transfusing patients according to accepted and evidence‐based transfusion thresholds.

To develop rigorous evidence‐based guidelines, according to the AGREE II checklist 2, systematic literature searches should form the basis of the guidelines. The methodology to conduct these systematic reviews, including an assessment in the certainty of the body of evidence, and the translation of the evidence into clinical, evidence‐based recommendations can be performed using the GRADE (Grading of Recommendations, Assessment, Development and Evaluations) approach.

GRADE is a transparent framework for developing and presenting summaries of evidence and provides a systematic approach for making clinical practice recommendations 3, 4, 5. To bridge the gap between research and clinical practice, the engagement of a multidisciplinary expert panel is key. Formal group consensus methods have been developed to organize subjective judgements and to synthesize them with the available evidence 6.

The National Institutes of Health (NIH) has developed a consensus development conference format to evaluate biomedical technologies and practices and to disseminate these results to health professionals and the public 6, 7. NIH consensus development conferences include participation by speakers who present the evidence, an audience that has the opportunity to comment on the evidence, and a panel that deliberates and produces a written statement based on its judgement 8. The strengths of the NIH consensus development process are its potential to translate a large body of evidence into practical clinical policy, to bring together apparently conflicting viewpoints, with the evidence as the “common denominator”, to draw public as well as professional attention to important clinical issues, to obtain front‐line practitioner input on the feasibility of evidence‐generated clinical policy and to increase the exposure of all parties to the existing research evidence in an area.^9^.

A diverse, international group of policymakers, managers, their support staff and other stakeholders indicated that the systematic consideration of the best available evidence would help to improve health system decision‐making processes 10. To improve the trustworthiness of claims about the effects of treatments and to formulate evidence‐based clinical recommendations, the systematic and transparent use of a methodological approach/framework and a formal consensus methodology is highly recommended 11, 12.

Therefore, in order to enhance shared decision‐making and to formulate evidence‐based recommendations in three PBM topics (preoperative anaemia, red blood cell (RBC) transfusion thresholds and implementation of PBM programmes), an international consortium of European, American, Canadian and Australian organizations in the field of blood transfusion organized a 2‐day (24 and 25 April 2018) International Consensus Conference on Patient Blood Management in Frankfurt/Main, Germany (ICC‐PBM 2018). The key results and conclusions/recommendations of this meeting were published elsewhere 13. The aim of this methodology paper was to report the ICC‐PBM 2018 proceedings and provide more insight how evidence‐based methodologies and technologies were used to enhance shared decision‐making when formulating clinical recommendations. This information will inform and guide all stakeholders involved in making and using evidence‐based recommendations.

## Materials & methods

A Scientific Committee was established consisting of 23 members including one methodologist from the Centre for Evidence‐Based Practice, Belgian Red Cross (Belgium) with expertise in conducting systematic reviews and 22 subject matter experts that were appointed by the following sponsoring and participating organizations: German Red Cross Blood Transfusion Services (Germany); Grenoble University Hospital (France); National Health Services Blood & Transplant (United Kingdom); Academic Hospital of Brest (France); European Blood Alliance (The Netherlands); Canadian Blood Services (Canada); Western Health, Melbourne (Australia); Fairview Health Services and Patient Readiness Institute, Minneapolis (USA); Ludwig Boltzmann Institute for Clinical and Experimental Traumatology, Vienna (Austria); One Blood, Orlando (USA); Institute for Immunology and Transfusion Medicine, Greifswald (Germany); Sanquin, Amsterdam (The Netherlands); Duke University School of Medicine, North Carolina (USA); Italian National Institute of Health (Italy); University Hospital Frankfurt, Frankfurt am Main (Germany); Institute for Transfusion Medicine and Immunohaematology, Frankfurt (Germany); Australian Red Cross Blood Service (Australia); Mount Sinai Hospital, Toronto (Canada); Établissement Français du Sang (France); Italian Society for Transfusion Medicine and Immunohaematology (Italy), Monash University, Melbourne (Australia) (Appendix 1, all appendices can be found in the online publication). The Scientific Committee was responsible to prepare the ICC‐PBM 2018 by selecting the 3 PBM topics of interest: (1) diagnosis and management of preoperative anaemia, (2) the use of RBC transfusion thresholds and (3) the implementation of PBM programmes.

### Patient Blood Management topics of interest and 17 PICO questions

After the face‐to‐face kick‐off meeting in February 2017, the Scientific Committee decided to select three PBM topics and formulated 17 PICO questions ((defining Population, Intervention, Comparison group, Outcome(s)) accordingly: (1) the diagnosis and management of preoperative anaemia (3 PICO questions), (2) the use of RBC transfusion thresholds (11 PICO questions) and (3) the implementation of PBM programmes (3 PICO questions). The 17 PICO questions were related to (1) definition (PICO 1), diagnosis (PICO 2) and management (PICO 3) of preoperative anaemia in adult elective surgery patients, (2) the use of RBC transfusion thresholds in intensive care and acute interventions (PICO 4‐9, & PICO 14), haematology and oncology (PICO 10 & PICO 11) and neurology (PICO 12 & PICO 13) and (3) the implementation of ‘comprehensive’ PBM programmes (PICO 15) and behavioural interventions (PICO 16) or decision support systems (PICO 17) to promote the implementation of PBM programmes. Detailed information about the PICO questions, selection criteria and corresponding search strategies are published elsewhere.^13^.

### Evidence‐based methodology

A stepwise, rigorous and transparent process was used to conduct evidence reviews starting from the formulation of a PICO question and selection criteria followed by developing search strategies in 4 different databases (MEDLINE (PubMed interface), EMBASE (via Embase.com), the Cochrane Library and the Transfusion Evidence Library). The following steps included screening of relevant papers (first on title and abstract, then on full text), data extraction, quality assessment and data synthesis (meta‐analyses if possible). The evidence reviews were performed by one methodologist (per review) from the Centre for Evidence‐Based Practice (Belgian Red Cross), and revisions were done by the Scientific Committee.

Detailed methodological principles to conduct the evidence reviews in the context of a clinical guideline project are published in a methodological charter 14. The GRADE approach was used to rate the certainty of evidence in the reviews and to translate the evidence from the reviews to conditional/weak, strong or research recommendations 5.

Starting from the PICO questions and the selection of outcomes, the Scientific Committee members independently assessed the relative importance of outcomes numerically on a 1–9 scale (7–9, critical; 4–6 important; and 1–3, of limited importance). Ranking outcomes by their relative importance can help guideline developers to focus attention, when formulating recommendations, on those outcomes that are considered most important 15. This rating exercise was performed after reviewing the evidence (February 2018) and detailed information can be found in Appendix 2. About 60–70% of the Scientific Committee members completed the rating scores for all outcomes related to the PICO questions of preoperative anaemia (*n* = 16, 70%), RBC transfusion thresholds (*n* = 16, 70%) and PBM implementation (*n* = 14, 61%). Based on the calculation of the mean rating scores and a discussion within the Scientific Committee in case of discrepant results (i.e. large variability in rating scores), final rating scores were provided.

A standard evidence summary template (in Word) was used to describe the PICO question, the search strategies, the search date, the selection criteria, the characteristics of included studies, the synthesis of findings, the quality of the evidence, the certainty of the body of evidence, the conclusion(s) and the references of the included studies 14. Additionally, evidence profiles were created with the GRADEpro software (https://gradepro.org/) to enable a summary of findings including an estimate of effect for each outcome and a quality rating of the evidence for each outcome according to the 8 GRADE criteria (five criteria that might potentially downgrade the quality: risk of bias, inconsistency, indirectness, imprecision, publication bias; and three criteria that might potentially upgrade the quality: large effect, dose–response and confounders).^3^

When developing recommendations, the GRADE approach recommends that an expert panel should use the evidence reviews as the fundamental source of information (including the quality of the evidence and the importance of outcomes) and recommends that an expert panel should subsequently formulate a conditional/weak of strong recommendation (strength) for or against an intervention (direction). In order to formulate appropriate recommendations, several items need to be considered such as the balance between benefits and harms, the quality of the evidence, values and preferences, resource use and the feasibility, equity and acceptability. These items are collected in an Evidence‐to‐Decision (EtD) framework (available in the GRADEpro software) which facilitates this consideration and enhances the formulation of recommendations in a clear and transparent manner 12, 16. Table 1 represents the 10 items and judgement questions of the EtD framework that were used as a central thread to formulate recommendations during the ICC‐PBM 2018. In general, the different evidence reviews answered whether a specific intervention is effective (or not). Therefore, the source of information for EtD items one (desirable effects), two (undesirable effects), three (certainty of evidence) and five (balance of effects) was the evidence reviews. The other items (4, 6, 8, 9, 10) were not ignored but were introduced and discussed in the different sessions and indicative opinion polls were organized (see below). The Scientific Committee decided to exclude item 7 (cost‐effectiveness) from the discussion because this item is too context‐ and healthcare system‐specific.

**Table 1:** GRADE’s Evidence‐to‐Decision framework used during the ICC‐PBM 2018

ITEMS (introduced by the chairs in the open sessions)	JUDGEMENT questions (answered by the decision‐making panels during the private sessions)	Source of information	Additional considerations
1. Desirable effects	How substantial are the desirable anticipated effects? Trivial Small Moderate Large Varies Don't know	Presentation of evidence reviews (including evidence profiles created by the GRADEpro software) by the Scientific Committee	The rapporteurs directly inserted the additional considerations by the general audience in the EtD template in word or in the GRADEpro software
2. Undesirable effects	How substantial are the undesirable anticipated effects? Large Moderate Small Trivial Varies Don't know	Presentation of evidence reviews (including evidence profiles created by the GRADEpro software) by the Scientific Committee	The rapporteurs directly inserted the additional considerations by the general audience in the EtD template in word or in the GRADEpro software
3. Certainty of evidence	What is the overall certainty of the evidence of effects? Very low Low Moderate High No included studies	Presentation of evidence reviews (including evidence profiles created by the GRADEpro software) by the Scientific Committee	The rapporteurs directly inserted the additional considerations by the general audience in the EtD template in word or in the GRADEpro software
4. Values	Is there important uncertainty about or variability in how much people value the main outcomes? Important uncertainty or variability Possibly important uncertainty or variability Probably no important uncertainty or variability No important uncertainty or variability	Opinion poll voting with the general audience via Mentimeter™, http://www.menti.com, Stockholm, Sweden	The rapporteurs directly inserted the additional considerations by the general audience in the EtD template in word or in the GRADEpro software
5. Balance of effects	Does the balance between desirable and undesirable effects favour the intervention or the comparison? Favours the comparison Probably favours the comparison Does not favour either the intervention or the comparison Probably favours the intervention Favours the intervention Varies Don't know	Presentation of evidence reviews (including evidence profiles created by the GRADEpro software) by the Scientific Committee	The rapporteurs directly inserted the additional considerations by the general audience in the EtD template in word or in the GRADEpro software
6. Resources required	How large are the resource requirements (costs)? Large costs Moderate costs Negligible costs and savings Moderate savings Large savings Varies Don't know	Opinion poll voting with the general audience via Mentimeter™, http://www.menti.com, Stockholm, Sweden	The rapporteurs directly inserted the additional considerations by the general audience in the EtD template in word or in the GRADEpro software
7. cost‐effectiveness	Does the cost‐effectiveness of the intervention favour the intervention or the comparison? Favours the comparison Probably favours the comparison Does not favour either the intervention or the comparison Probably favours the intervention Favours the intervention Varies No included studies	The Scientific Committee decided to exclude this item from the discussion because too context‐ and healthcare system‐specific	
8. Equity	What would be the impact on health equity? Reduced Probably reduced Probably no impact Probably increased Increased Varies Don’t know	Opinion poll voting with the general audience via Mentimeter™, http://www.menti.com, Stockholm, Sweden	The rapporteurs directly inserted the additional considerations by the general audience in the EtD template in word or in the GRADEpro software
9. Acceptability	Is the intervention acceptable to key stakeholders? No Probably no Probably yes Yes Varies Don't know	Opinion poll voting with the general audience via Mentimeter™, http://www.menti.com, Stockholm, Sweden	The rapporteurs directly inserted the additional considerations by the general audience in the EtD template in word or in the GRADEpro software
10. Feasibility	Is the intervention feasible to implement? No Probably no Probably yes Yes Varies Don't know	Opinion poll voting with the general audience via Mentimeter™, http://www.menti.com, Stockholm, Sweden	The rapporteurs directly inserted the additional considerations by the general audience in the EtD template in word or in the GRADEpro software

### Formal consensus methodology

The use of the NIH consensus development conference methodology resulted in the following four consecutive major steps:

1. At day 1, evidence reviews were presented by Scientific Committee members in three parallel public (open) sessions (according to the three selected topics) followed by discussion with the general audience. The general audience was divided over these three sessions (according to their preference which was recorded during the registration for the conference): 46 people attended the ‘preoperative anaemia’ session, 68 people attended the session on ‘RBC transfusion thresholds’ and 61 people attended the ‘PBM implementation’ session. Each session was chaired by two experts (one subject matter expert and one methodologist), and two Scientific Committee members (per session) served as the Rapporteurs by recording the key points from the discussion with the general audience. Before the ICC‐PBM 2018, the speakers, chairs and rapporteurs were trained in the GRADE methodology and the Consensus Conference methodology via an online webinar. A detailed list of the speakers, chairs and rapporteurs can be found in Appendix 3. After having the evidence reviews presented by different speakers (mainly Scientific Committee members), the chairs introduced the nine Evidence‐to‐Decision framework items to the audience: desirable effects (item 1), undesirable effects (item 2), certainty of the evidence (item 3), values (item 4), balance of effects (item 5), resources required (item 6), equity (item 8), acceptability (item 9), feasibility (item 10). These items served as the central themes during the discussion with the audience. Items 1, 2, 3 and 5 were covered by the evidence reviews which were used as the fundamental source of information. The remaining items (items 4, 6, 8, 9, 10; item 7 was not discussed cfr. supra) were also part of the discussion and indicative opinion polls of the audience were collected via the Mentimeter™ software (http://www.menti.com, Stockholm, Sweden, application via smartphone or laptop). We summarized the results from the indicative opinion polls by expressing a range (%) of different answers from one or more items. Since these opinion polls were not binding but rather indicative for the decision‐making panels, no consensus definition was used. The rapporteurs used the GRADEpro software to insert the additional considerations by the general audience directly into the EtD framework (Table 1).
2. At the end of day 1, further deliberation on the evidence and additional considerations by the general audience was done by the three decision‐making panels (one panel per topic) during three separate and closed (executive) sessions. The decision‐making panels consisted of 10–15 subject matter experts (including 2–3 Scientific Committee members) together with the same chairs (one subject matter expert and one methodologist) as for the open sessions. Key information from the evidence reviews together with important additional considerations of the general audience was presented via the EtD framework (directly in the GRADEpro software) by the chairs after which discussion among the decision‐making panellists was started and judgements on the different EtD items were completed. The two rapporteurs (per session) recorded the judgement decisions into the summary of judgement template of the GRADEpro software. Finally, based on this summary of judgements, a conclusion was drafted including the formulation of a conditional, strong or research recommendation (using standard wording) together with the underlying justifications.
3. At day 2, a general plenary session with all conference attendees was organized and moderated by three chairs (Appendix 3). During this session, the chairs of the open/parallel sessions (day 1) presented the summary of judgements of the EtD framework together with the draft recommendations and major justifications. Subsequently, the general audience was able to give their indicative opinion poll vote (using the Mentimeter™ software) on these draft recommendations to identify the level of agreement (i.e. ‘accept completely’, ‘accept with some reservation’, ‘accept with major reservation’, ‘reject with reservation’, ‘reject completely’) for each recommendation. Finally, plenary discussion was held and recorded by the rapporteurs.
4. At the end of day 2, three separate and final closed (executive) sessions were organized with the decision‐making panels. Based on the plenary session presentations (i.e. GRADE’s EtD framework), discussions with the general audience and indicative opinion poll votes on the draft recommendations, changes to the draft recommendations were made (if needed) and final recommendations and justifications were formulated. Consensus among the decision‐making panellists was defined as a two‐thirds majority (via hand raising).

## Results

**Figure 1:**
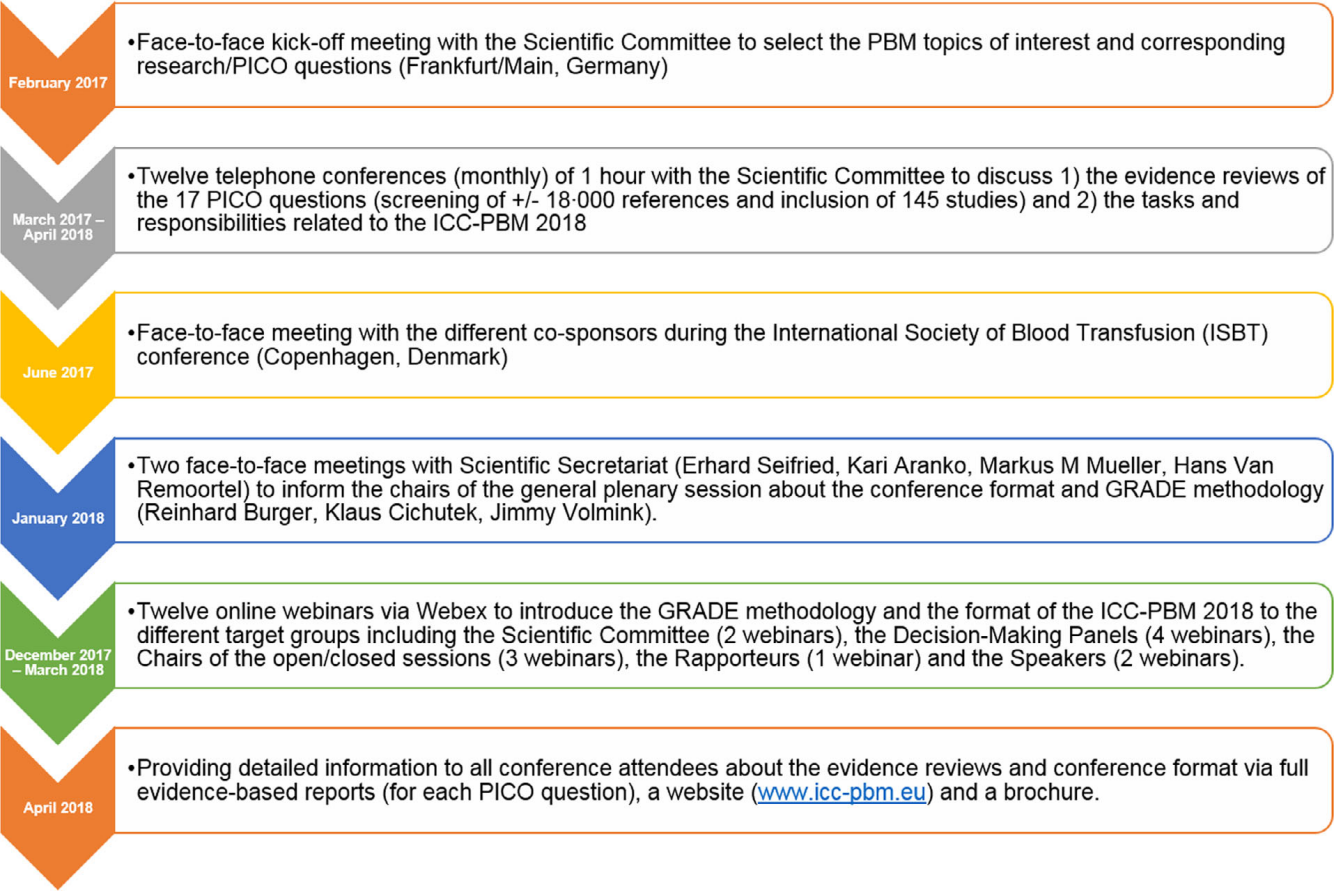
Overview of the key tasks to prepare the ICC‐PBM.

A total number of 186 persons from five continents and 35 countries were registered for the 2‐day Conference (Appendix 4). The attendees represented different medical disciplines, including transfusion medicine, clinical haematology, pathology, anaesthesiology, oncology, cardiology, neurology, surgery, and critical care medicine and methodological expertise in conducting systematic reviews. The attendees were affiliated to 63 (University/Academic) Hospitals, 28 Blood Services, 23 (Patient) Organizations, 12 (Pharmaceutical) Companies and 5 Governmental Bodies. All contributing Institutions are listed in Appendix 5. The different co‐sponsors of the ICC‐PBM were acknowledged followed by a brief introduction of the three PBM topics, and the composition of the three corresponding decision‐making panels tasked to formulate evidence‐based recommendations were presented. A detailed list of these decision‐making panels can be found in Appendix 6. Figure 1 summarizes how shared decision‐making was promoted by the use of the Consensus Development Conference format, the GRADE’s EtD framework/software and indicative opinion polls via a smartphone/laptop application (Mentimeter^TM^ software).

### Day 1: three parallel sessions and a draft consensus statement

The screening of 17 607 references resulted in the final inclusion of 145 studies 13. In the ‘preoperative anaemia’ session, the evidence of 62 studies regarding the definition and diagnosis of preoperative anaemia (PICOs 1–2: 36 observational studies) and the treatment of preoperative anaemia in adult elective surgery patients (PICO 3: three observational studies and 23 experimental studies) were presented. The mean response rate of the indicative opinion poll was 56% (range 50–76%). About 55% of the respondents decided that there is (probably) no important uncertainty or variability in how people/patients value the critical outcomes. About 40% of the people believe that prophylactic transfusion has a reduced impact on health equity whereas only 12–21% of the respondents felt that this reduction is present in case of iron monotherapy or ESA+iron therapy, respectively. The majority of people (50–70%) said that prophylactic transfusion is (probably) not acceptable nor feasible to key stakeholders whereas ~60–80% mentioned that iron monotherapy or ESA+iron therapy is (probably) acceptable and feasible.

The ‘RBC transfusion thresholds’ session started with an introductory keynote presentation by Prof. Dr. Jeffrey L. Carson (Robert Wood Johnson Medical School, Rutgers University, New Brunswick, NJ, USA) followed by the presentation of the evidence from 40 studies that investigated the effectiveness of a more restrictive transfusion threshold compared to a more liberal transfusion threshold in the following adult patient populations: critically ill but clinically stable intensive care (PICO 4: four experimental studies), orthopaedic and non‐cardiac surgery (PICO 5: 11 experimental studies), acute (gastrointestinal) bleeding (PICOs 6 and 14: four experimental studies), symptomatic coronary heart disease (PICO 7: two experimental studies), septic shock (PICO 8: two experimental studies), cardiac surgery (PICO 9: eight experimental studies), haematology (PICO 10: two experimental studies), oncology (PICO 11: three experimental studies), neurology (PICOs 12 and 13: one observational study and two experimental studies).

The mean response rate of the indicative opinion poll was 81% (range 77–82%). About 82% of the respondents decided that there is (probably) important uncertainty or variability in how people/patients value the critical outcomes. Fifty‐four per cent indicated that the use of restrictive RBC transfusion thresholds will result in negligible costs/savings to moderate/large savings. About 60% of the people believe that the use of restrictive RBC transfusion thresholds will probably have no impact or even an increased impact on health equity. The majority of people (70‐90%) said that restrictive RBC transfusion strategies are probably acceptable and feasible to key stakeholders.

In the ‘PBM implementation’ session, the evidence of 43 studies regarding the implementation of comprehensive PBM programmes (PICO 15: 20 observational studies) and potential behavioural interventions (PICO 16: 19 observational studies) or computerized decision support systems (PICO 17: 3 observational studies and one experimental study) was presented.

Detailed information of all evidence reviews and presentations is available at the ICC‐PBM website 17. The mean response rate of the indicative opinion poll was 65% (range 56–80%). About 60% of the respondents decided that there is (probably) important uncertainty or variability in how people/patients value the critical outcomes. About half of the people believe that the impact of PBM programmes on health equity varies. The majority of people (63–85%) said that behavioural interventions and decision support systems are probably acceptable and feasible to key stakeholders. Detailed information about the indicative opinion polls can be downloaded from the ICC‐PBM website 17.

During the second part of the three parallel sessions, a closed/executive session with the decision‐making panels took place. Based on the evidence reviews, the additional considerations and the indicative polling of the audience, the decision‐making panels made final judgements on the different EtD criteria. A summary of judgements for the 17 PICO questions can be found in Appendix 7. The draft recommendations/conclusions of the decision‐making panels can be found in Appendix 8.

### Day 2: general plenary session and a final consensus statement

The draft recommendations and underlying justifications from the decision‐making panels were presented to the general audience. Detailed information of these presentations and the indicative opinion poll results is available at the ICC‐PBM website.^17^ The median response rate of the indicative opinion poll on these draft recommendations was 68 [IQR: 10]%. In summary, the majority of the audience accepted the draft recommendations completely of the diagnosis and management of preoperative anaemia (74 [IQR: 7]%), the use RBC transfusion thresholds (81 [IQR: 16]%) and PBM implementation (69 [IQR: 6]%) completely, except one recommendation which was accepted completely by only ~20% of the audience. This recommendation was related to the management of anaemia and the use of ESAs in addition to iron supplementation in adult preoperative elective major orthopaedic surgery patients with haemoglobin levels <13 g/dl. Major concerns from the audience were that this recommendation did cover a (too) big number of different anaemia causes and it does not cover the caveats/contraindications and the costs of ESAs. It was also mentioned that clinical outcomes should be considered as the critical outcomes rather than reduction in RBC transfusion since it only replaces one treatment by another. Because transfusion rate in orthopaedic surgery dropped dramatically over the past years, the included older studies overestimate the effect of ESA+iron therapy on RBC reduction and may make the recommendation obsolete. The audience also commented that the avoidance of RBC transfusion reported in published studies depends on the transfusion rate (number of patients transfused), which is dependent on the time of publication, patient population and disease/operation planned. Finally, the audience indicated that ethical considerations, patient preferences, financial and equity consideration are key aspects when formulating this recommendation.

At the end of the second day, the decision‐making panels formulated final recommendations. For preoperative anaemia, four clinical and three research recommendations were formulated, including a strong recommendation to detect and manage anaemia sufficiently early before major elective surgery. For RBC transfusion thresholds, four clinical and six research recommendation were developed, including two strong clinical recommendations for critically ill but clinically stable intensive care patients with or without septic shock (recommended threshold for RBC transfusion, haemoglobin concentration <7 g/dl) as well as for patients undergoing cardiac surgery (recommended threshold for RBC transfusion, haemoglobin concentration <7.5 g/dl). For implementation of PBM programmes, two clinical and three research recommendations were developed, including recommendations to implement comprehensive PBM programmes and to use electronic decision support systems (both conditional recommendations) to improve appropriate RBC utilization. Detailed information about the final recommendations is published elsewhere.^13^ **.**

## Discussion

Numerous consensus conference formats have been used to address issues in medical care 18. The most well‐developed consensus conference methodology has been used by the US National Institutes of Health (NIH) Consensus Conference format 19. Consensus conferences are designed to minimize the ability of individual opinions to dominate a group discussion and limit the influence of other social pressures and constructs. These features may be particularly relevant when the topics under discussion are contentious.

For the first time in the field of PBM, and after two years of preparation, clinical bedside experts from anaesthesiology, haemostasis and thrombosis, intensive care medicine, transfusion medicine, internal medicine, neurology, clinical laboratory medicine, haematology, clinical immunology, oncology, neurosurgery, vascular, cardiac and oncological surgery, gynaecology and obstetrics met with nurses, patient and blood banking representatives, representatives of blood transfusion services and government authorities, Evidence‐Based Medicine methodologists as well as epidemiologists for a two‐day intensive exchange guided by the GRADE methodology. Prior to the meeting, significant deliberation was put into the framing of the questions to be addressed. Consistent with the NIH model, the ICC‐PBM relied on a broad‐based independent panel to bring balanced perspectives and relevant knowledge to the process. In addition to subject matter experts, both practitioners and methodologists as well as representatives of blood recipients participated. Information was presented in an open meeting format with opportunity for both panellists and audience to question and discuss. Lastly, draft recommendations (consensus statement) were generated at the end of the first day and presented to the entire group of participants on the second day for discussion and refinement. Because of the use of the GRADE methodology, the ICC‐PBM spent less time on expert presentations than is typical for most NIH Consensus Conferences. Consensus conferences are dependent on acceptance of the scientific method and a robust application of scientific rigour generates trust in the process. By thoroughly training all members of the Scientific Committee, all panellists, chairs, rapporteurs and speakers in the GRADE and EtD methodologies as well as in the use of the GRADEpro software by establishing more than ten webinars in addition to the regular teleconferences before the conference, we were able to facilitate the whole process. All critical partners had a deep and thorough methodology knowledge before attending the ICC. Furthermore, use of GRADE methodology, including a thorough assessment of the relevant literature, provided a solid foundation for consideration of the PICO questions.

Together with the active involvement of the audience comprising about another 150 clinical experts, the three panels evaluated the outcome of the literature search performed following the predefined PICO questions. The panellists were able to agree on the above‐mentioned recommendations in the three predefined PBM topics on the diagnosis and treatment of preoperative anaemia, RBC transfusion thresholds and the implementation of PBM programmes 13.

The GRADE and EtD methodology enabled the three panels to integrate evidence‐based literature searches into a discussion on practical bedside implementation and guided the drafting of evidence‐based recommendations. Employing the tools described above to maintain academic rigour through the process, the three panels were able to take a holistic view of PBM. One of the major outcomes of this process are the recommendations, which go beyond the simple interpretation of the published literature to give concise advice to the bedside clinician and to patients potentially requiring RBC transfusion, ESA and/or iron support while keeping in line with the published evidence.

Several challenges or limitations regarding the use of the Consensus Conference format and the GRADE approach were present. Firstly, although an enormous effort was made to inform and educate all people involved in the evidence‐based methodology (i.e. GRADE approach), the formulation of straightforward PICO questions and selection criteria, including rating the importance of outcomes, was a difficult discussion between the methodologists and the subject matter experts. This is part of the lumping–splitting debate where subject matter experts are more lumping the review questions, that is addressing a wide range of populations, interventions and outcomes. Lumped review questions are time‐consuming but will better inform decisions about which interventions to implement when there may be a range of options. On the other hand, methodologists are more in favour of splitting the review questions, that is addressing a narrow range of populations, interventions and outcomes. These type of questions are less time‐consuming. However, these reviews can only inform decisions about whether or not to implement narrowly focused interventions. Given the tight time schedule (i.e. 1 year) to complete the 17, predominantly lumped evidence reviews, in addition to the technical preparation of the ICC‐PBM 2018, we might have missed some relevant studies (selection bias). Therefore, a face‐to‐face meeting at the beginning of the project with the formulation and final approval of clinically relevant and feasible PICO questions (given the available time and resources) together with the preparation of an a priori data analysis plan should have been better to conduct evidence reviews in the most efficient way. To further improve the engagement of the Scientific Committee members, the decision‐making panels, chairs and rapporteurs, a second online/face‐to‐face meeting with the Scientific Committee, the rapporteurs and the decision‐making panels could be organized after conducting the evidence reviews to discuss the results, critical appraisal and the conclusions of these evidence reviews. Secondly, the formulation of the recommendations is the key task for decision‐making panels. Therefore, a rigorous procedure is required to select panellists that accept the principles of Evidence‐Based Medicine and to manage potential conflicts of interest in a fair, judicious and transparent manner 20. Where substantial disagreement on the formulation of recommendation exists (i.e. ICC‐PBM recommendation on the use of ESAs and iron in preoperative major orthopaedic surgery patients with Hb <13 g/dl), a formal and blinded voting system among the decision‐making panels (e.g. via the GRADEpro software) could be helpful to avoid the (negative) impact of biased/strong individual opinions. Thirdly, to maximize participation and minimize the costs, consensus conferences could be held in conjunction with other educational or scientific meetings.

## Conclusion

The use of a formal Consensus Conference format in combination with the GRADE approach provides a powerful framework to formulate evidence‐based recommendations based on the best available evidence, patient perspectives and clinical expertise. This first international consensus conference on PBM could open ways to future developments in this field. First, it provides a tool to help evaluate the outcomes from this consensus conference (e.g. changes in clinical practices, published clinical studies according to the ICC‐PBM recommendations). Second, the emitted recommendations should be reviewed and updated at least every 5 years according to the same methodology. The systematic use of evidence‐based methodologies should be the new standard to evaluate (cost‐) effectiveness of medical treatments in order to avoid recommendations based on expert opinion solely and/or strongly held opinions on religious (e.g. Jehovah Witnesses) or commercial (e.g. pharmaceutical companies) grounds. Third, future consensus conferences using the same methodology should be organized to elaborate other recommendations in areas not covered by the first ICC‐PBM such as PBM for platelet and plasma transfusion or PBM in paediatrics or obstetrics.

## Conflict of interests

Dr Van Remoortel reported receiving financial support/funding to his institution from organizers of the ICC‐PBM 2018 (see list of co‐sponsors listed for Dr Mueller below) during the conduct of the study. Dr Mueller reported receiving grants from the European Blood Alliance (EBA), American Association of Blood Banks (AABB), International Society of Blood Transfusion (ISBT), Deutsche Gesellschaft für Transfusionsmedizin und Immunhämatologie (DGTI), Societe Francaise de Transfusion Sanguine (SFTS) and Societa Italiana di Medicina Trasfusionale e Immunoematologia (SIMTI) during the conduct of the study and receiving personal fees from TerumoBCT outside the submitted work. Dr De Buck reported receiving financial support/funding to her institution from organizers of the ICC‐PBM 2018 (see list of co‐sponsors listed for Dr Mueller above) during the conduct of the study. Dr Devine reported receiving grants from Macopharma, TerumoBCT and Hemanext outside the submitted work. Dr Meybohm reported receiving grants, personal fees and nonfinancial support from B. Braun Melsungen, CSL Behring, Fresenius Kabi, and Vifor Pharma; receiving personal fees from Pharmacosmos outside the submitted work; and receiving research grants from the German Research Foundation (ME 3559/1‐1, ME 3559/3‐1), International Anesthesia Research Society, German Society of Anaesthesiology and Intensive Care Medicine, and European Society of Anaesthesiology. Dr Wood reported receiving grants from Celgene Corporation, CSL Behring, Australian Red Cross Blood Service, New Zealand Blood Service, Amgen, Abbvie, Bristol‐Myers Squibb, Janssen, Roche, Sanofi and Takeda outside the submitted work. Dr Seifried received personal fees from Vivor outside the submitted work and reported receiving grants from the European Blood Alliance (EBA), American Association of Blood Banks (AABB), International Society of Blood Transfusion (ISBT), Deutsche Gesellschaft für Transfusionsmedizin und Immunhämatologie (DGTI), Societe Francaise de Transfusion Sanguine (SFTS) and Societea Italiana di Medicina Transfusionale e Immunoematologia (SIMTI) during the conduct of the study. No other authors reported disclosures.

**Figure 2:**
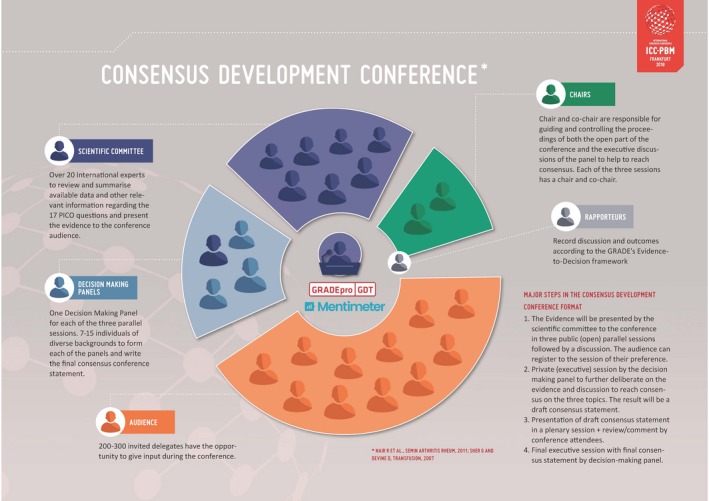
Promoting shared decision‐making in an international guideline project: the use of the Consensus Development Conference format, GRADE’s Evidence‐to‐Decision framework and software (GRADEpro) and opinion polls via smartphone or laptop application (Mentimeter^TM^ software).

## Supporting information


**Appendix S1 **Overview of the 23 Scientific Committee Members.
**Appendix S2 **Rating the importance of outcomes for all PICO questions by the Scientific Committee members
**Appendix S3 **Chairs, presenters and rapporteurs of the three parallel sessions (day 1) and the general plenary session (day 2)
**Appendix S4 **List of continents and countries included in the participation list of the ICC‐PBM 2018
**Appendix S5 **List of Institutions/Organizations that co‐sponsored or contributed during the ICCPBM 2018
**Appendix S6 **Composition decision‐making panels
**Appendix S7 **Summary of judgements of the Evidence‐to‐Decision framework items relevant to the 17 PICO questions
**Appendix S8 **Draft recommendations of the decision‐making panels at the end of day 1
