# Supplementary material for: The systematic use of evidence‐based methodologies and technologies enhances shared decision‐making in the 2018 International Consensus Conference on Patient Blood Management
Source: Vox Sang. 2019 Nov 10;115(1):60–71. doi: 10.1111/vox.12852 (PMC7004058; doi:10.1111/vox.12852)
Supplement: Supplementary file 1 — Appendix S1 Overview of the 23 Scientific Committee Members. Appendix S2 Rating the importance of outcomes for all PICO questions by the Scientific Committee members Appendix S3 Chairs, presenters and rapporteurs of the three parallel sessions (day 1) and the general plenary session (day 2) Appendix S4 List of continents and countries included in the participation list of the ICC‐PBM 2018 Appendix S5 List of Institutions/Organizations that co‐sponsored or contributed during the ICCPBM 2018 Appendix S6 Composition decision‐making panels Appendix S7 Summary of judgements of the Evidence‐to‐Decision framework items relevant to the 17 PICO questions Appendix S8 Draft recommendations of the decision‐making panels at the end of day 1 [file VOX-115-60-s001.pdf]

Appendix 1 Overview of the 23 Scientific Committee Members

Name	Affiliation
Erhard Seifried (chair)	German Red Cross Blood Transfusion Services (Germany)
Pierre Albaladejo	Grenoble University Hospital (France)
Shubha Allard	NHS Blood & Transplant (United Kingdom)
Cécile Aubron	Academic Hospital of Brest (France)
Kari Aranko	European Blood Alliance (The Netherlands)
Dana Devine	Canadian Blood Services (Canada)
Craig French	Western Health, Melbourne (Australia)
Kathrine P Frey	Fairview Health Services and Patient Readiness Institute, Minneapolis (USA)
Christian Gabriel	Ludwig Boltzmann Institute for Clinical and Experimental Traumatology, Vienna (Austria)
Richard Gammon	One Blood, Orlando (USA)
Andreas Greinacher	Institute for Immunology and Transfusion Medicine, Greifswald (Germany)
Marian van Kraaij	Sanquin, Amsterdam (The Netherlands)
Jerrold Levy	Duke University School of Medicine, North Carolina (USA)
Giancarlo Liumbruno	Italian National Institute of Health (Italy)
Patrick Meybohm	University Hospital Frankfurt, Frankfurt am Main (Germany)
Markus M Mueller	Institute for Transfusion Medicine and Immunohaematology, Frankfurt (Germany)
Michael F Murphy	NHS Blood & Transplant (United Kingdom)
Ben Saxon	Australian Red Cross Blood Service (Australia)
Nadine Shehata	Mount Sinai Hospital, Toronto (Canada)
Pierre Tiberghien	Établissement Français du Sang (France)
Hans Van Remoortel	Centre for Evidence-Based Practice, Belgian Red Cross (Belgium)
Claudio Velati	Italian Society for Transfusion Medicine and Immunohaematology (Italy)
Erica M Wood	Monash University, Melbourne (Australia)

Appendix 2 Rating the importance of outcomes for all PICO questions by the Scientific Committee members

rating scale:								
1	2	3	4	5	6	7	8	9
of **least** importance								of **most** importance
of limited importance for making a decision (not included in evidence profile)			important, but not critical for making a decision (included in evidence profile)			Critical for making a decision (included in evidence profile)		

PICO question	Outcome	Rating scores on a 1-9 scale by Scientific Committee Members (initials)																				
		AG	PM	KF	MiM	JL	NS	CA	DD	GL	RG	EW	CF	ES	MM	BS	CG	MEAN	MEDIAN	MINIMUM	MAXIMUM	Final rating (after discussion)
PICO 1	Mortality: 30-day mortality	8	8	8	8	9	9	9	8	[illegible]	9	9	6	9	9	9	7	8	9	6	9	Critical
	Mortality: in-hospital mortality	8	8	8	8	9	9	7	7	[illegible]	9	9	6	9	9	7	9	8	8	6	9	Critical
	Acute myocardial infarction	8	5	8	5	9	9	8	8	[illegible]	9	9	6	8	8	8	9	8	8	5	9	Critical
	Acute ischaemic stroke	6	4	8	4	9	9	7	7	[illegible]	9	9	6	8	8	8	9	7	8	4	9	Critical
	Acute kidney injury	6	7	8	7	9	9	7	7	[illegible]	5	9	3	8	8	8	4	7	7	3	9	Crtical
	Acute mesenteric ischemia	6	4	5	4	9	9	6	7	[illegible]	4	4	3	8	8	6	4	6	6	3	9	Important
	Acute peripheral vascular ischemia	4	4	5	4	9	9	6	7	[illegible]	9	4	3	8	8	6	3	6	6	3	9	Important
																		MEAN	MEDIAN	MINIMUM	MAXIMUM	
PICO 3	Mortality: (all-cause) mortality	8	4	8	6	9	9	9	7	9	9	9	7	9	9	9	8	8	9	4	9	Critical
	Acute myocardial infarction	7	4	8	6	9	9	8	7	9	9	9	5	8	8	8	9	8	8	4	9	Critical
	Acute ischaemic stroke	7	4	8	4	9	9	7	7	8	9	9	5	8	8	8	9	7	8	4	9	Critical
	Acute kidney injury	5	4	8	6	9	9	7	7	8	5	9	5	8	8	8	4	7	7,5	4	9	Critical
	Acute mesenteric ischaemia	4	4	6	4	9	9	6	7	8	4	4	5	8	8	6	3	6	6	3	9	Important
	Acute peripheral vascular ischaemia	4	4	6	4	9	9	6	7	8	5	4	5	8	8	6	3	6	6	3	9	Important
	Length of hospital stay	4	5	7	5	9	6	6	8	9	9	7	3	4	4	6	7	6	6	3	9	Important
	Any type of reported infection	4	7	7	7	9	4	7	7	4	9	7	6	7	7	7	7	7	7	4	9	Important
	RBC utilization	7	8	8	8	9	4	6	9	9	9	7	6	2	2	4	7	7	7	2	9	Important
	Thromboembolic events	4	5	6	5	9	7	7	7	7	9	7	2	6	6	6	3	6	6	2	9	Critical

PICO question	Outcome	Rating scores on a 1-9 scale by Scientific Committee Members (initials)																				
		AG	PM	KF	MiM	JL	NS	CA	DD	GL	RG	EW	CF	ES	MM	PT	BS	MEAN	MEDIAN	MINIMUM	MAXIMUM	Final rating (after discussion)
PICO 4-14	30-day mortality	9	7	9	9	9	9	9	9	9	9	9	6	9	9	9	9	9	9	6	9	Critical
	90-day mortality	7	9	8	9	7	7	9	8	3	5	9	6	9	9	8	8	8	8	3	9	Critical
	hospital mortality	7	7	9	7	8	9	6	8	9	9	7	6	9	9	9	8	8	8	6	9	Critical
	1-year mortality	4	5	6	7	6	4	6	6	3	5	6	6	9	9	6	7	6	6	3	9	Important
	mortality at the time of longest follow-up	3	8	7	8	3	4	4	7	3	3	5	8	7	7	5	7	6	6	3	8	Important
	participants exposed to blood transfusion	6	9	9	9	8	6	6	7	9	7	5	7	2	2	3	4	6	6,5	2	9	Important
	units of blood transfused	6	7	8	7	9	5	6	6	9	7	5	4	2	2	4	3	6	6	2	9	Important
	haemoglobin concentration	5	5	[illegible]	5	8	4	1	7	9	9	3	2	4	4	4	5	5	5	1	9	Important
	cardiac events	8	5	6	5	5	9	7	6	8	9	7	4	8	8	6	8	7	7	4	9	Critical
	myocardial infarction	8	7	7	7	6	9	9	6	8	9	7	4	8	8	6	7	7	7	4	9	Critical
	congestive heart failure	6	5	6	5	9	8	6	5	6	9	5	4	8	8	5	7	6	6	4	9	Critical
	sepsis-bacteraemia	6	5	7	5	9	6	7	6	6	9	6	4	8	8	4	7	6	6	4	9	Important
	pneumonia	5	5	7	5	8	6	7	5	5	9	6	2	8	8	3	6	6	6	2	9	Important
	pneumonia or wound infection	5	5	7	5	8	6	7	6	6	5	6	2	8	8	4	6	6	6	2	8	Important
	limb ischaemia	4	4	4	4	5	8	4	4	3	2	6	2	8	8	3	6	5	4	2	8	Important
	ICU readmission	5	4	5	4	5	6	4	7	8	2	6	2	8	8	[illegible]	4	5	5	2	8	Important
	ICU length of stay	4	6	7	6	5	3	2	6	8	2	6	2	6	6	[illegible]	7	5	6	2	8	Important
	days to first transfusion	3	3	3	3	5	2	2	3	7	2	3	2	2	2	3	3	3	3	2	7	Not important
	sever adverse reaction	8	5	6	5	9	4	7	5	9	2	7	5	8	8	4	7	6	6,5	2	9	Important
	any adverse event related to transfusion	4	9	7	9	9	4	6	4	9	5	7	5	8	8	4	7	7	7	4	9	Critical
	days with fever	3	7	3	7	4	3	3	3	3	2	6	2	4	4	4	5	4	3,5	2	7	Not important
	pulmonary edema or respiratory distress	4	8	7	8	9	6	7	5	6	9	7	2	8	8	4	6	7	7	2	9	Critical
	rash	3	2	3	2	6	2	2	3	3	2	3	2	2	2	3	3	3	2,5	2	6	Not important
	hypotension	3	2	3	2	5	6	3	3	3	2	6	2	3	3	3	6	3	3	2	6	Not important
	symptomatic vasospasm	3	2	3	2	3	6	2	4	3	2	3	4	4	4	2	4	3	3	2	6	Not important
	transfusion-related hemolysis (acute or delayed)	8	7	8	7	9	4	3	5	6	2	6	2	8	8	3	6	6	6	2	9	Important
	transfusion-related fever	4	5	7	5	6	2	3	4	3	2	6	2	4	4	3	3	4	4	2	7	Important
	transfusion-related allergy with urticaria	4	4	7	4	6	2	4	4	3	2	1	2	2	2	3	4	3	3,5	1	7	Not important
	transfusion-related pulmonary edema	7	4	8	4	9	6	6	5	7	2	6	2	8	8	4	7	6	6	2	9	Important
	transfusion-related viral infection	7	7	8	7	9	3	6	4	3	2	3	2	8	8	4	7	6	6,5	2	9	Important
	transfusion-related new alloantibodies	3	7	7	7	6	4	4	3	7	2	3	2	6	6	4	6	5	5	2	7	Important
	number of chemotherapy cycles	3	2	3	2	5	[illegible]	4	4	3	2	1	2	1	1	1	5	3	2	1	5	Not important
	duration of chemotherapy	2	2	3	2	5	[illegible]	4	3	3	2	1	2	1	1	1	3	2	2	1	5	Not important
	chemotherapy-related neutropenia	2	2	3	2	6	[illegible]	3	3	3	2	1	2	1	1	1	6	3	2	1	6	Not important
	chemotherapy-related neutropenic infection	2	2	3	2	6	[illegible]	7	3	3	2	4	2	1	1	3	6	3	3	1	7	Not important
	chemotherapy-related thrombocytopenia	2	3	3	3	6	[illegible]	2	3	3	2	3	2	4	4	3	6	3	3	2	6	Not important
	chemotherapy-related fatigue	2	3	3	3	3	6	7	3	3	2	6	5	1	1	3	4	3	3	1	7	Not important
	chemotherapy-related nausea and vomiting	2	3	3	3	3	3	2	3	3	2	1	2	1	1	1	2	2	2	1	3	Not important
	chemotherapy-related oral mucositis	2	3	3	3	3	3	2	3	3	2	1	2	1	1	2	2	2	2	1	3	Not important
	chemotherapy-related diarrhea	2	3	3	3	2	3	2	3	3	2	1	2	1	1	1	2	2	2	1	3	Not important
	chemotherapy-related constipation	2	3	3	3	1	[illegible]	2	3	3	2	1	2	1	1	1	2	2	2	1	3	Not important
	complications from RBC transfusions	7	9	7	7	9	6	7	5	9	5	7	5	8	8	4	7	7	7	4	9	Critical
	CVA-stroke	8	5	7	5	7	8	7	4	7	5	7	2	8	8	3	8	6	7	2	8	Critical
	any cerebral infarction on MRI	8	5	7	5	9	6	7	4	7	5	4	2	8	8	3	8	6	6,5	2	9	Important
	delayed cerebral infarction	7	5	7	5	6	[illegible]	7	3	6	3	6	2	8	8	2	8	6	6	2	8	Important
	ARDS/ALI	9	8	7	8	9	7	7	4	7	2	7	2	8	8	4	8	7	7	2	9	Critical
	thromboembolism	6	7	7	7	6	7	7	3	7	4	6	2	8	8	3	7	6	7	2	8	Critical
	renal failure	6	7	7	7	6	8	7	4	7	5	6	2	8	8	2	7	6	7	2	8	Critical
	rebleeding	7	5	6	5	8	8	7	4	9	5	6	2	6	6	4	7	6	6	2	9	Important
	episodes of neutropenic fever	2	3	3	3	3	3	7	3	3	2	4	2	1	1	4	4	3	3	1	7	Not important
	need for mechanical ventilation	6	3	6	3	4	6	6	4	6	2	7	2	7	7	3	7	5	6	2	7	Important
	need for inotropic agents	4	4	4	4	6	6	6	4	6	2	7	2	7	7	2	5	5	4,5	2	7	Important
	renal replacement therapy	6	5	5	5	6	7	7	4	4	2	7	2	7	7	3	5	5	5	2	7	Important
	mental confusion	7	3	3	3	4	6	6	3	4	2	5	2	6	6	4	4	4	4	2	7	Important
	function and fatigue (EQ-5D)	6	4	5	4	6	4	8	4	6	9	7	6	5	5	5	5	6	5	4	9	Important
	SF-36: physical component summary score	5	4	5	4	6	4	7	[illegible]	4	2	7	6	5	5	[illegible]	5	5	5	2	7	Important
	SF-36: mental component summary score	5	4	5	4	6	4	7	[illegible]	4	2	6	6	5	5	[illegible]	5	5	5	2	7	Important
	Health-related quality of life (EQ-5D) at 6 weeks	8	5	5	5	6	4	7	4	4	5	6	6	7	7	5	7	6	5,5	4	8	Important
	Health-related quality of life (EQ-5D) at 3 months	4	5	5	5	4	4	7	4	4	5	6	6	7	7	3	7	5	5	3	7	Important
	inability to walk or death at 30 days	8	6	6	6	9	4	7	5	9	9	7	6	8	8	[illegible]	7	7	7	4	9	Critical
	inability to walk or death at 60 days	5	8	6	8	9	4	7	5	6	9	7	6	8	8	[illegible]	7	7	7	4	9	Critical
	lower extremity physical activities of daily living at 30 days	5	5	6	5	3	4	7	4	8	9	4	6	6	6	4	4	5	5	3	9	Important
	lower extremity physical activities of daily living at 60 days	4	6	6	6	2	3	7	4	6	9	4	6	6	6	4	4	5	6	2	9	Important
	instrumental activities of daily living at 30 days	7	5	5	5	5	4	7	5	8	9	4	6	6	6	3	6	6	5,5	3	9	Important
	instrumental activities of daily living at 60 days	5	4	5	4	5	3	7	5	6	9	4	5	6	6	3	6	5	5	3	9	Important
	energy/fatigue at 30 days	6	4	6	4	3	4	7	4	8	9	6	5	4	4	5	4	5	4,5	3	9	Important
	energy/fatigue at 60 days	4	4	6	4	3	3	7	4	6	9	6	5	4	4	5	4	5	4	3	9	Important
	fatigue scale score	4	4	6	4	3	4	7	5	6	5	6	2	4	4	4	5	5	4	2	7	Important
	timed up and go test	4	4	5	4	3	4	6	5	4	2	6	2	4	4	[illegible]	4	4	4	2	6	Important
	length of inpatient stay	4	4	7	4	8	4	4	6	9	2	6	2	6	6	3	4	5	4	2	9	Important

PICO question	Outcome	Rating scores on a 1-9 scale by Scientific Committee Members (initials)																		
		AG	PM	KF	MiM	JL	NS	CA	DD	RG	EW	CF	ES	MM	BS	MEAN	MEDIAN	MINIMUM	MAXIMUM	Final rating (after discussion)
PICO 15	Blood product utilization - number of patients receiving PLT transfusion	6	5	7	5	9	4	9	4	2	9	7	8	8	8	7	7	2	9	Critical
	Blood product utilization - number of patients receiving RBC transfusion	7	7	7	7	9	6	9	7	9	9	7	6	6	8	7	7	6	9	Critical
	Blood product utilization - number of patients receiving FFP transfusion	5	6	7	6	9	5	9	3	2	9	7	8	8	6	7	7	2	9	Critical
	Blood product utilization - number of patients receiving cryoprecipitate transfusion	5	3	6	3	9	5	6	3	2	9	7	8	8	8	6	6	2	9	Important
	Blood product utilization - number of patients receiving any transfusion	4	7	7	7	9	4	7	4	9	9	7	6	6	8	7	7	4	9	Critical
	Blood product utilization - number of RBC units transfused	7	7	7	7	9	4	9	6	9	7	7	6	6	7	7	7	4	9	Critical
	Blood product utilization - number of FFP units transfused	5	6	7	6	9	4	9	3	2	7	7	8	8	4	6	7	2	9	Critical
	Blood product utilization - number of PLT units transfused	5	6	7	6	9	4	9	3	2	7	7	8	8	4	6	7	2	9	Critical
	Blood product utilization - number of cryoprecipitate units transfused	4	3	6	3	9	4	6	3	2	7	7	8	8	6	6	6	2	9	Important
	Blood product utilization - number of any blood products transfused	3	6	7	6	9	5	9	4	2	7	7	8	8	6	6	7	2	9	Critical
	Morbidity - composite measures	3	7	7	7	9	9	7	6	9	7	2	6	6	4	7	7	2	9	Critical
	Morbidity - acute myocardial infarction	5	7	7	7	9	9	7	6	9	9	2	7	7	4	7	7	2	9	Critical
	Morbidity - acute ischaemic stroke	5	7	7	7	9	9	7	6	9	9	2	7	7	4	7	7	2	9	Critical
	Morbidity - acute kidney injury	5	7	7	7	9	9	7	7	9	9	2	7	7	4	7	7	2	9	Critical
	Mortality - hospital mortality	5	7	7	7	9	9	7	6	9	9	6	9	9	4	8	7	4	9	Critical
	Mortality - 30-day mortality	5	7	7	7	9	9	7	6	9	9	6	9	9	4	8	7	4	9	Critical
	Mortality - not specified	3	7	7	7	6	9	7	3	9	6	6	9	9	6	7	7	3	9	Critical
	Length of hospital stay	3	6	7	7	9	6	6	6	9	7	2	6	6	6	6	6	2	9	Important
PICO 16	Blood product utilization - number of patients receiving PLT transfusion	4	7	7	7	9	4	9	6	2	9	7	8	8	8	7	7	2	9	Critical
	Blood product utilization - number of patients receiving RBC transfusion	4	7	7	7	9	6	9	9	9	9	7	6	6	8	8	7	6	9	Critical
	Blood product utilization - number of patients receiving FFP transfusion	4	7	7	7	9	5	9	5	2	9	7	8	8	6	7	7	2	9	Critical
	Blood product utilization - number of patients receiving cryoprecipitate transfusion	4	2	6	2	9	5	6	5	2	9	7	8	8	8	6	6	2	9	Important
	Blood product utilization - number of patients receiving any transfusion	4	6	7	6	9	4	9	7	5	9	7	6	6	8	7	7	4	9	Critical
	Blood product utilization - number of RBC units transfused	8	7	7	7	9	4	9	7	9	7	2	6	6	7	7	7	2	9	Critical
	Blood product utilization - number of FFP units transfused	6	7	7	7	9	4	9	4	2	7	2	8	8	4	6	7	2	9	Critical
	Blood product utilization - number of PLT units transfused	6	7	7	7	9	4	9	5	2	7	2	8	8	4	6	7	2	9	Critical
	Blood product utilization - number of cryoprecipitate units transfused	4	3	6	3	9	4	6	4	2	7	2	8	8	6	5	6	2	9	Important
	Blood product utilization - number of any blood products transfused	4	7	7	7	9	5	7	4	5	7	2	8	8	7	6	7	2	9	Critical
PICO 17	Blood product utilization - number of patients receiving PLT transfusion	5	7	7	7	9	`	9	6	5	9	3	6	6	8	7	7	3	9	Critical
	Blood product utilization - number of patients receiving RBC transfusion	7	7	7	7	9	`	9	9	5	9	3	8	8	8	7	8	3	9	Critical
	Blood product utilization - number of patients receiving FFP transfusion	5	7	7	7	9	`	9	6	5	9	3	8	8	6	7	7	3	9	Critical
	Blood product utilization - number of patients receiving cryoprecipitate transfusion	4	3	6	3	9	`	6	5	4	9	3	8	8	8	6	6	3	9	Important
	Blood product utilization - number of patients receiving any transfusion	4	7	7	7	9	`	7	7	9	9	3	6	6	8	7	7	3	9	Critical
	Blood product utilization - number of RBC units transfused	8	7	7	7	9	`	9	5	9	7	3	6	6	7	7	7	3	9	Critical
	Blood product utilization - number of FFP units transfused	6	7	7	7	9	`	9	4	9	7	3	8	8	6	7	7	3	9	Critical
	Blood product utilization - number of PLT units transfused	6	7	7	7	9	`	9	4	9	7	3	8	8	7	7	7	3	9	Critical
	Blood product utilization - number of cryoprecipitate units transfused	4	2	6	2	9	`	6	4	9	7	3	8	8	7	6	6,5	2	9	Important
	Blood product utilization - number of any blood products transfused	4	6	7	6	9	`	7	5	9	7	3	8	8	7	7	7	3	9	Critical
	Transfusion-related, transfusion-transmitted infection, transfusion-associated circulatory overload, transfusion-associated dyspnea, acute transfusion reactions	7	7	7	7	9	7	8	7	3	7	2	9	9	7	7	7	2	9	Critical
	Bleeding (including WHO grade 3 or 4, or equivalent or bleeding that requires an operation)	7	4	3	4	9	9	6	8	5	7	2	9	9	7	6	7	2	9	Important
	Infection	4	6	6	6	9	6	7	7	5	6	2	9	9	6	6	6	2	9	Important
	Arterial or venous thromboembolism (including deep vein thrombosis, pulmonary embolism, stroke, myocardial infarction)	7	5	7	5	9	8	7	6	5	7	2	9	9	7	7	7	2	9	Critical
	Number of transfusions compliant with institutional transfusion guidelines	8	5	9	5	9	4	7	8	9	7	7	6	6	6	7	7	4	9	Critical
	Blood count or coagulation parameter (e.g. haematocrit, haemoglobin, prothrombin time, partial thromboplastin time, or platelet count) preceding and after the transfusion.	4	4	7	4	9	4	6	8	9	7	2	6	6	6	6	6	2	9	Important
	Length of participant stay (in-hospital)	4	5	7	5	9	6	6	6	9	7	3	8	8	7	7	7	3	9	Critical
	Length of participant stay (ICU)	4	4	7	4	9	6	6	6	5	7	3	8	8	7	6	6	3	9	Important
	All-cause mortality	9	7	7	7	9	9	7	5	9	7	7	9	9	7	8	7	5	9	Critical
	Clinician workflow (additional time per intervention implemented)	7	4	6	4	5	3	6	3	3	7	6	4	4	3	4	4	3	7	Important

Appendix 3 Chairs, presenters and rapporteurs of the three parallel sessions (day 1) and the general plenary session (day 2)

Parallel session 1 (day 1): 'diagnosis and management preoperative anaemia'

Name	Role	Affiliation
Emmy De Buck	Chair	Centre for Evidence-Based Practice (CEBaP), Belgian Red Cross, Mechelen, Belgium; Department of Public Health and Primary Care, Faculty of Medicine, KU Leuven, Leuven, Belgium
Yves Ozier	Chair	Departments of Anaesthesiology and Critical Care Medicine, University Hospital of Brest, Brest, France
Kathrine P Frey	Presenter	Fairview Health Services and Patient Readiness Institute, Minneapolis, MN, U.S.A.
Katerina Pavenski	Presenter	St. Michael´s Hospital and University of Toronto, Toronto, Canada
Patrick Meybohm	Rapporteur	Department of Anaesthesiology, Intensive Care Medicine and Pain Therapy, University Hospital Frankfurt, Frankfurt/Main, Germany
Markus M Mueller	Rapporteur	German Red Cross Blood Transfusion Service, Frankfurt/Main, Germany

Parallel session 2 (day 1): 'RBC transfusion triggers'

Name	Role	Affiliation
Reinhard Burger	Chair	Robert Koch Institute, Berlin (Germany)
Jimmy Volmink	Chair	Department of Clinical Epidemiology, Faculty of Medicine and Health Sciences, Stellenbosch University, Stellenbosch, South Africa
Cécile Aubron	Presenter	Departments of Anaesthesiology and Critical Care Medicine, University Hospital of Brest, Brest, France
Jeffrey L Carson	Presenter	Robert Wood Johnson Medical School, Rutgers University, New Brunswick, NJ, U.S.A.
Richard Gammon	Presenter	One Blood, Orlando, FL, U.S.A.
Jerrold Levy	Presented	Department of Cardiothoracic Intensive Care Medicine, Duke University Medical Centre, Durham, NC, U.S.A.
Cynthia So-Osman	Panel member	Sanquin Blood Bank, Leiden and Department of Haematology, Groene Hart Hospital, Gouda, The Netherlands; International Society of Blood Transfusion (ISBT), Amsterdam, The Netherlands
Gilles Folléa	Rapporteur	Société Française de Transfusion Sanguine (SFTS), Paris, France
Erica Wood	Rapporteur	Transfusion Research Unit, Department of Epidemiology and Preventive Medicine, Monash University, Melbourne, Australia; International Society of Blood Transfusion (ISBT), Amsterdam, The Netherlands

Parallel session 3 (day 1): 'implementation of PBM programmes

Name	Role	Affiliation
Dean Fergusson	Chair	Departments of Medicine, Surgery, Epidemiology and Public Health, University of Ottawa, Ottawa, Canada
Jonathan Waters	Chair	Departments of Anaesthesiology and Bioengineering, University of Pittsburgh Medical Centre, Pittsburgh, PA, U.S.A.
Mike Murphy	Presenter	National Health Service Blood & Transplant and University of Oxford, Oxford, U.K.
Dana Devine	Rapporteur	Canadian Blood Services, Ottawa, Canada
Pierre Tiberghien	Rapporteur	Etablissement Français du Sang (EFS), Saint-Denis, France

Plenary session (day 2): presentation draft recommendation to the general audience

Name	Role	Affiliation
Reinhard Burger	Chair	Robert Koch Institute, Berlin (Germany)
Jimmy Volmink	Chair	Department of Clinical Epidemiology, Faculty of Medicine and Health Sciences, Stellenbosch University, Stellenbosch, South Africa
Klaus Cichutek	Chair	Paul-Ehrlich-Institute (PEI), Langen, Germany
Emmy De Buck	Presenter	Centre for Evidence-Based Practice (CEBaP), Belgian Red Cross, Mechelen, Belgium; Department of Public Health and Primary Care, Faculty of Medicine, KU Leuven, Leuven, Belgium
Yves Ozier	Presenter	Departments of Anaesthesiology and Critical Care Medicine, University Hospital of Brest, Brest, France
Craig French	Presenter	Intensive Care, Western Health, Melbourne, Australia
Dean Fergusson	Presenter	Departments of Medicine, Surgery, Epidemiology and Public Health, University of Ottawa, Ottawa, Canada
Jonathan Waters	Presenter	Departments of Anaesthesiology and Bioengineering, University of Pittsburgh Medical Centre, Pittsburgh, PA, U.S.A.

Appendix 4 List of continents and countries included in the participation list of the ICC-PBM 2018

Continent	Countries
Europe	Austria, Belgium, Croatia, Denmark, Estonia, Finland, France, Germany, Hungary, Ireland, Italy, Malta, the Netherlands, Portugal, Romania, Slovenia, Spain, Switzerland, United Kingdom, Sweden
Asia	Bahrain, China, Hong Kong, Lebanon, Oman, the Philippines, Saudi Arabia, Thailand
Africa	Egypt, Libya, Morocco, South Africa
The Americas	United States of America, Canada
Oceania	Australia

Appendix 5 List of Institutions/Organizations that co-sponsored or contributed during the ICC-PBM 2018

Co-sponsors	Contributors
The American Association of Blood Banks (AABB), the International Society of Blood Transfusion (ISBT), the German Society of Transfusion Medicine and Immunohaematology (DGTI), the French Society of Blood Transfusion (SFTS), the Italian Society of Transfusion Medicine and Immunohaematology (SIMTI) and the European Blood Alliance (EBA)	Australian Red Cross Blood Service (ARCBS), the Canadian Blood Services (CBS), the International Collaboration for Transfusion Medicine Guidelines (ICTMG), the International Society on Thrombosis and Haemostasis (ISTH), the National Blood Authority in Australia (NBA), the Austrian Society for Blood Group Serology and Transfusion Medicine (ÖGBT), French Society of Anaesthesia and Critical Care SFAR, the World Health Organization (WHO), the European Commission, the German Society of Anaesthesiology and Intensive Care Medicine (DGAI), the National Health Authority Australia and the Paul-Ehrlich-Institute (Langen, Germany).

Appendix 6 Composition decision-making panels

Decision-making panel 'diagnosis and management preoperative anaemia'

Name	Role	Affiliation
Emmy De Buck	Chair	Centre for Evidence-Based Practice, Belgian Red Cross (Belgium)
Yves Ozier	Chair	University Hospital of Brest (France)
Danielle Bischof	Panel member	Mt Sinai Hospital, Toronto (Canada)
Christian Gabriel	Panel member	Ludwig Boltzmann Institute for Clinical and Experimental Traumatology, Vienna (Austria)
Jennifer Hamilton	Panel member	Patient Representative (USA)
Sigismond Lasocki	Panel member	University Hospital, Angers (France)
Manuel Muñoz Gomez	Panel member	University of Malaga (Spain)
Thomas Schmitz-Rixen	Panel member	Goethe-University Hospital Frankfurt am Main (Germany)

Table (Continued)

Name	Role	Affiliation
Hubert Serve	Panel member	University of Frankfurt (Germany)
Amanda Thomson	Panel member	Australian Red Cross Blood Service (Australia)
Claudio Velati	Panel member	Italian Society of Transfusion Medicine and Immunohaematology (Italy)
Agneta Wikman	Panel member	Karolinska University, Stockholm (Sweden)
Patrick Meybohm	Rapporteur	University Hospital Frankfurt, Frankfurt am Main (Germany)
Markus M Mueller	Rapporteur	German Red Cross Transfusion Service, Frankfurt/Main (Germany)

Decision-making panel 'RBC transfusion triggers'

Name	Role	Affiliation
Reinhard Burger	Chair	Robert Koch Institute, Berlin (Germany)
Jimmy Volmink	Chair	University of Stellenbosch (South Africa)
Pierre Albaladejo	Panel member	University Hospital, Grenoble (France)
Erik Beckers	Panel member	University Medical Center, Maastricht (The Netherlands)
Kaaron Benson	Panel member	Moffitt Cancer Center, Tampa, Florida (USA)
Jeffrey Carson	Panel member	Rutgers University, New Jersey (USA)
Graham Donald	Panel member	Patient Representative, (United Kingdom)
Craig French	Panel member	Western Health, Melbourne (Australia)
Nicole Juffermans	Panel member	University of Amsterdam (The Netherlands)
Marian van Kraaij	Panel member	Sanquin, Amsterdam (The Netherlands)
Dawn Maze	Panel member	University of Toronto (The Netherlands)
Marek Mirski	Panel member	Johns Hopkins Medical Institutions, Baltimore (USA)
Gavin Murphy	Panel member	British Heart Foundation & University of Leicester (United Kingdom)
Jean-Jacques Ries	Panel member	University Hospital, Basel (Switzerland)
Ben Saxon	Panel member	Australian Red Cross Blood Service (Australia)
Christof Sohn	Panel member	University of Heidelberg (Germany)
Tim Walsh	Panel member	University of Edinburgh (UK)
Gilles Folléa	Rapporteur	French Society of Blood Transfusion, Paris (France)
Erica Wood	Rapporteur	International Society of Blood Transfusion & Monash University, Melbourne (Australia)

Decision-making panel 'implementation of PBM programmes'

Name	Role	Affiliation
Dean Fergusson	Chair	University of Ottawa (Canada)
Jonathan Waters	Chair	University of Pittsburgh (USA)
Shubha Allard	Panel member	NHS Blood & Transplant (United Kingdom)
Lauren Anthony	Panel member	Allina Health, Minneapolis (USA)
Linley Bielby	Panel member	Australian Red Cross Blood Service (Australia)
Lise Estcourt	Panel member	NHS Blood & Transplant (United Kingdom)
Mohamed El Missiry	Panel member	Patient Representative, Charité Hospital, Berlin (Germany)
Steven Frank	Panel member	Johns Hopkins Medical Institutions, Baltimore (USA)
John Freedman	Panel member	St Michael's Hospital, Toronto (Canada)
Catherine Humbrecht	Panel member	Établissement Français du Sang, Strasbourg (France)
Giancarlo Liumbruno	Panel member	Italian National Institute of Health (Italy)
Louise Sherliker	Panel member	NHS Blood & Transplant (United Kingdom)
Hans Van Remoortel	Panel member	Centre for Evidence-Based Practice, Belgian Red Cross (Belgium)
Dana Devine	Rapporteur	Canadian Blood Services, Ottawa (Canada)
Pierre Tiberghien	Rapporteur	Établissement Français du Sang, Saint-Denis (France)

Appendix 7 Summary of judgements of the Evidence-to-Decision framework items relevant to the 17 PICO questions

PICO 1: In elective surgery patients [Population], is preoperative anaemia [Intervention/Risk factor] a risk factor for adverse clinical or economic outcomes [Outcome] compared to no preoperative anaemia [Comparison]?

PICO 2: In elective surgery preoperative patients [Population], should Hb of 130 g/L (Index test) (versus [comparator test] [Comparison]) be used to diagnose anaemia [Outcome]?

Domain	Judgement						
Desirable effects	**Trivial**	Small	Moderate	Large		Varies	Don't know
Undesirable effects	Large	**Moderate**	Small	Trivial		Varies	Don't know
Certainty OF evidence	Very low	**Low**	Moderate	High			No included studies
Values	Important uncertainty or variability	Possibly important uncertainty or variability	Probably no important uncertainty or variability	**No important uncertainty or variability**			
Balance of effects	**Favours the comparison**	Probably favours the comparison	Does not favour either the intervention or the comparison	Probably favours the intervention	Favours the intervention	Varies	Don't know

PICO 3 (intervention of interest: prophylactic transfusion): In elective surgery patients with preoperative anaemia [Population], is the use of red blood cell transfusion [Intervention] effective to improve clinical and economic outcomes [Outcome] compared to no intervention/placebo/standard of care [Comparison]?

Domain	Judgement						
Desirable effects	**Trivial**	Small	Moderate	Large		Varies	Don't know
Undesirable effects	Large	Moderate	Small	**Trivial**		Varies	Don't know
Certainty of evidence	**Very low**	Low	Moderate	High			No included studies
Values	Important uncertainty or variability	Possibly important uncertainty or variability	Probably no important uncertainty or variability	**No important uncertainty or variability**			
Balance of effects	Favours the comparison	Probably favours the comparison	**Does not favour either the intervention or the comparison**	sProbably favours the intervention	Favours the intervention	Varies	Don't know
Resources required	Large costs	**Moderate costs**	Negligible costs and savings	Moderate savings	Large savings	Varies	Don't know
Cost-effectiveness	Favours the comparison	Probably favours the comparison	Does not favour either the intervention or the comparison	Probably favours the intervention	Favours the intervention	Varies	**No included studies**
Equity	Reduced	**Probably reduced**	Probably no impact	Probably increased	Increased	Varies	Don't know
Acceptability	**No**	Probably no	Probably yes	Yes		Varies	Don't know
Feasibility	No	Probably no	Probably yes	**Yes**		Varies	Don't know

PICO 3 (intervention of interest: iron monotherapy): In elective surgery patients with preoperative anaemia [Population], is the use of iron monotherapy [Intervention] effective to improve clinical and economic outcomes [Outcome] compared to no intervention/placebo/standard of care [Comparison]?

Domain	Judgement						
Desirable effects	Trivial	Small	Moderate	**Large**		Varies	Don't know
Undesirable effects	Large	Moderate	**Small**	Trivial		Varies	Don't know
Certainty of evidence	Very low	**Low**	Moderate	High			

Table (Continued)

Domain	Judgement						
							No included studies
Values	Important uncertainty or variability	Possibly important uncertainty or variability	Probably no important uncertainty or variability	**No important uncertainty or variability**			
Balance of effects	Favours the comparison	Probably favours the comparison	Does not favour either the intervention or the comparison	**Probably favours the intervention**	Favours the intervention	Varies	Don't know
Resources required	Large costs	Moderate costs	Negligible costs and savings	Moderate savings	Large savings	**Varies**	Don't know
Cost-effectiveness	Favours the comparison	Probably favours the comparison	Does not favour either the intervention or the comparison	Probably favours the intervention	Favours the intervention	Varies	**No included studies**
Equity	Reduced	Probably reduced	Probably no impact	**Probably increased**	Increased	Varies	Don't know
Acceptability	No	Probably no	Probably yes	**Yes**		Varies	Don't know
Feasibility	No	Probably no	**Probably yes**	Yes		Varies	Don't know

PICO 3 (intervention of interest: ESA monotherapy): In elective surgery patients with preoperative anaemia [Population], is the use of erythrocyte stimulating agents [Intervention] effective to improve clinical and economic outcomes [Outcome] compared to no intervention/placebo/standard of care [Comparison]?

Domain	JudgEment						
Desirable Effects	Trivial	Small	**Moderate**	Large		Varies	Don't know
Undesirable Effects	Large	Moderate	Small	**Trivial**		Varies	Don't know
Certainty of evidence	**Very low**	Low	Moderate	High			No included studies
Values	Important uncertainty or variability	Possibly important uncertainty or variability	Probably no important uncertainty or variability	**No important uncertainty or variability**			
Balance of effects	Favours the comparison	Probably favours the comparison	Does not favour either the intervention or the comparison	Probably favours the intervention	Favours the intervention	Varies	**Don't know**
Resources required	Large costs	Moderate costs	Negligible costs and savings	Moderate savings	Large savings	**Varies**	Don't know
Cost-effectiveness	Favours the comparison	Probably favours the comparison	Does not favour either the intervention or the comparison	Probably favours the intervention	Favours the intervention	Varies	**No included studies**
Equity	Reduced	Probably reduced	Probably no impact	Probably increased	Increased	Varies	**Don't know**
Acceptability	No	Probably no	Probably yes	Yes		Varies	**Don't know**
Feasibility	No	Probably no	Probably yes	Yes		Varies	**Don't know**

PICO 3 (intervention of interest: ESA + iron therapy): In elective surgery patients with preoperative anaemia [Population], is the use of red blood cell transfusion or iron supplementation and/or erythrocyte stimulating agents [Intervention] effective to improve clinical and economic outcomes [Outcome] compared to no intervention/placebo/standard of care [Comparison]?

Domain	Judgement						
Desirable effects	Trivial	Small	**Moderate**	Large		Varies	Don't know
Undesirable effects	Large	**Moderate**	Small	Trivial		Varies	Don't know
Certainty of evidence	Very low	**Low**	Moderate	High			No included studies
Values			Probably no important uncertainty or variability				

Table (Continued)

Domain	Judgement						
	Important uncertainty or variability	Possibly important uncertainty or variability		**No important uncertainty or variability**			
Balance of effects	Favours the comparison	Probably favours the comparison	**Does not favour either the intervention or the comparison**	Probably favours the intervention	Favours the intervention	Varies	Don't know
Resources required	Large costs	Moderate costs	Negligible costs and savings	Moderate savings	Large savings	Varies	**Don't know**
Cost-effectiveness	Favours the comparison	Probably favours the comparison	Does not favour either the intervention or the comparison	Probably favours the intervention	Favours tshe intervention	Varies	**No included studies**
Equity	Reduced	Probably reduced	Probably no impact	Probably increased	Increased	Varies	**Don't know**
Acceptability	No	Probably no	**Probably yes**	Yes		Varies	Don't know
Feasibility	No	Probably no	**Probably yes**	Yes		Varies	Don't know

PICO 4: In critically ill, but clinically stable adult intensive care patients [Population], is the use of a restrictive transfusion threshold [Intervention] effective to reduce mortality and improve other clinical outcomes [Outcome] compared to a liberal transfusion threshold [Comparison]?

Domain	Judgement						
Desirable effects	Trivial	Small	**Moderate**	Large		Varies	Don't know
Undesirable effects	Large	Moderate	Small	**Trivial**		Varies	Don't know
Certainty of evidence	Very low	Low	**Moderate**	High			No included studies
Values	**Important uncertainty or variability**	Possibly important uncertainty or variability	Probably no important uncertainty or variability	No important uncertainty or variability			
Balance of effects	Favours the comparison	Probably favours the comparison	Does not favour either the intervention or the comparison	**Probably favours the intervention**	Favours the intervention	Varies	Don't know
Resources required	Large costs	Moderate costs	Negligible costs and savings	Moderate savings	Large savings	**Varies**	Don't know
Cost-effectiveness	Favours the comparison	Probably favours the comparison	Does not favour either the intervention or the comparison	Probably favours the intervention	Favours the intervention	Varies	**No included studies**
Equity	Reduced	Probably reduced	Probably no impact	Probably increased	Increased	**Varies**	Don't know
Acceptability	No	Probably no	**Probably yes**	Yes		Varies	Don't know
Feasibility	No	Probably no	**Probably yes**	Yes		Varies	Don't know

PICO 5: In elderly high risk (cardiovascular) patients undergoing orthopaedic or non-cardiac surgery [Population], is the use of a restrictive transfusion threshold [Intervention] effective to reduce mortality and improve other clinical outcomes [Outcome] compared to a liberal transfusion threshold [Comparison]?

Domain	Judgement						
Desirable effects	Trivial	Small	**Moderate**	Large		Varies	Don't know
Undesirable effects	Large	Moderate	**Small**	Trivial		Varies	Don't know
Certainty of evidence	Very low	Low	**Moderate**	High			No included studies
Values	**Important uncertainty or variability**	Possibly important uncertainty or variability	Probably no important uncertainty or variability	No important uncertainty or variability			
Balance of effects						Varies	Don't know

Table (Continued)

Domain	Judgement						
	Favours the comparison	Probably favours the comparison	Does not favour either the intervention or the comparison	**Probably favours the intervention**	Favours the intervention		
Resources required	Large costs	Moderate costs	Negligible costs and savings	Moderate savings	Large savings	**Varies**	Don't know
Cost-effectiveness	Favours the comparison	Probably favours the comparison	Does not favour either the intervention or the comparison	Probably favours the intervention	Favours the intervention	Varies	**No included studies**
Equity	Reduced	Probably reduced	Probably no impact	Probably increased	Increased	**Varies**	Don't know
Acceptability	No	Probably no	**Probably yes**	Yes		Varies	Don't know
Feasibility	No	Probably no	Probably yes	**Yes**		Varies	Don't know

PICO 6: In patients with an acute gastrointestinal bleeding [Population], is the use of a restrictive transfusion threshold [Intervention] effective to reduce mortality and improve other clinical outcomes [Outcome] compared to a liberal transfusion threshold [Comparison]?

Domain	Judgement						
Desirable effects	Trivial	Small	**Moderate**	Large		Varies	Don't know
Undesirable effects	Large	Moderate	Small	**Trivial**		Varies	Don't know
Certainty of evidence	Very low	**Low**	Moderate	High			No included studies
Values	Important uncertainty or variability	**Possibly important uncertainty or variability**	Probably no important uncertainty or variability	No important uncertainty or variability			
Balance of effects	Favours the comparison	Probably favours the comparison	Does not favour either the intervention or the comparison	**Probably favours the intervention**	Favours the intervention	Varies	Don't know
Resources required	Large costs	Moderate costs	Negligible costs and savings	Moderate savings	Large savings	**Varies**	Don't know
Cost-effectiveness	Favours the comparison	Probably favours the comparison	Does not favour either the intervention or the comparison	Probably favours the intervention	Favours the intervention	Varies	**No included studies**
Equity	Reduced	Probably reduced	Probably no impact	Probably increased	Increased	**Varies**	Don't know
Acceptability	No	Probably no	**Probably yes**	Yes		Varies	Don't know
Feasibility	No	Probably no	Probably yes	**Yes**		Varies	Don't know

PICO 7: In patients with symptomatic coronary heart disease [Population], is the use of a restrictive transfusion threshold [Intervention] effective to reduce mortality and improve other clinical outcomes [Outcome] compared to a liberal transfusion threshold [Comparison]?

Domain	Judgement					
Desirable effects	Trivial	**Small**	Moderate	Large	Varies	Don't know
Undesirable effects	Large	**Moderate**	Small	Trivial	Varies	Don't know
Certainty of evidence	Very low	**Low**	Moderate	High		No included studies
Values	**Important uncertainty or variability**	Possibly important uncertainty or variability	Probably no important uncertainty or variability	No important uncertainty or variability		
Balance of effects					Varies	Don't know

Table (Continued)

**Domain**	**Judgement**						
	Favours the comparison	**Probably favours the comparison**	Does not favour either the intervention or the comparison	Probably favours the intervention	Favours the intervention		
Resources required	Large costs	Moderate costs	Negligible costs and savings	Moderate savings	Large savings	**Varies**	Don't know
Cost-effectiveness	Favours the comparison	Probably favours the comparison	Does not favour either the intervention or the comparison	Probably favours the intervention	Favours the intervention	Varies	**No included studies**
Equity	Reduced	Probably reduced	Probably no impact	Probably increased	Increased	**Varies**	Don't know
Acceptability	No	Probably no	**Probably yes**	Yes		Varies	Don't know
Feasibility	No	Probably no	Probably yes	**Yes**		Varies	Don't know

PICO 8: In patients with septic shock [Population], is the use of a restrictive transfusion threshold [Intervention] effective to reduce mortality and improve other clinical outcomes [Outcome] compared to a liberal transfusion threshold [Comparison]?

**Domain**	**Judgement**						
Desirable effects	Trivial	Small	**Moderate**	Large		Varies	Don't know
Undesirable effects	Large	Moderate	Small	**Trivial**		Varies	Don't know
Certainty of evidence	Very low	Low	**Moderate**	High			No included studies
Values	Important uncertainty or variability	Possibly important uncertainty or variability	Probably no important uncertainty or variability	No important uncertainty or variability			
Balance of effects	Favours the comparison	Probably favours the comparison	Does not favour either the intervention or the comparison	**Probably favours the intervention**	Favours the intervention	Varies	Don't know
Resources required	Large costs	Moderate costs	Negligible costs and savings	Moderate savings	Large savings	**Varies**	Don't know
Cost-effectiveness	Favours the comparison	Probably favours the comparison	Does not favour either the intervention or the comparison	Probably favours the intervention	Favours the intervention	Varies	No included studies
Equity	Reduced	Probably reduced	Probably no impact	Probably increased	Increased	**Varies**	Don't know
Acceptability	No	Probably no	**Probably yes**	Yes		Varies	Don't know
Feasibility	No	Probably no	**Probably yes**	Yes		Varies	Don't know

PICO 9: In patients undergoing cardiac surgery [Population], is the use of a restrictive transfusion threshold [Intervention] effective to reduce mortality and improve other clinical outcomes [Outcome] compared to a liberal transfusion threshold [Comparison]?

**Domain**	**Judgement**					
Desirable effects	Trivial	Small	**Moderate**	Large	Varies	Don't know
Undesirable effects	Large	Moderate	Small	**Trivial**	Varies	Don't know
Certainty of evidence	Very low	Low	**Moderate**	High		No included studies
Values	**Important uncertainty or variability**	Possibly important uncertainty or variability	Probably no important uncertainty or variability	No important uncertainty or variability		
Balance of effects					Varies	Don't know

Table (Continued)

Domain	Judgement						
	Favours the comparison	Probably favours the comparison	Does not favour either the intervention or the comparison	**Probably favours the intervention**	Favours the intervention		
Resources required	Large costs	Moderate costs	Negligible costs and savings	Moderate savings	Large savings	**Varies**	Don't know
Cost-effectiveness	Favours the comparison	Probably favours the comparison	Does not favour either the intervention or the comparison	Probably favours the intervention	Favours the intervention	Varies	No included studies
Equity	Reduced	Probably reduced	Probably no impact	Probably increased	Increased	**Varies**	Don't know
Acceptability	No	Probably no	**Probably yes**	Yes		Varies	Don't know
Feasibility	No	Probably no	**Probably yess**	Yes		Varies	Don't know

PICO 10: In adult haematological patients [Population], is the use of a restrictive transfusion threshold [Intervention] effective to reduce mortality and improve other clinical outcomes [Outcome] compared to a liberal transfusion threshold [Comparison]?

Domain	Judgement						
Desirable effects	**Trivial**	Small	Moderate	Large		Varies	Don't know
Undesirable effects	Large	Moderate	Small	**Trivial**		Varies	Don't know
Certainty of evidence	Very low	**Low**	Moderate	High			No included studies
Values	**Important uncertainty or variability**	Possibly important uncertainty or variability	Probably no important uncertainty or variability	No important uncertainty or variability			
Balance of effects	Favours the comparison	Probably favours the comparison	**Does not favour either the intervention or the comparison**	Probably favours the intervention	Favours the intervention	Varies	Don't know
Resources required	Large costs	Moderate costs	Negligible costs and savings	Moderate savings	Large savings	**Varies**	Don't know
Cost-effectiveness	Favours the comparison	Probably favours the comparison	Does not favour either the intervention or the comparison	Probably favours the intervention	Favours the intervention	Varies	**No included studies**
Equity	Reduced	Probably reduced	Probably no impact	Probably increased	Increased	**Varies**	Don't know
Acceptability	No	Probably no	**Probably yes**	Yes		Varies	Don't know
Feasibility	No	Probably no	**Probably yes**	Yes		Varies	Don't know

PICO 11: In adult patients with solid tumours [Population], is the use of a restrictive transfusion threshold [Intervention] effective to reduce mortality and improve other clinical outcomes [Outcome] compared to a liberal transfusion threshold [Comparison]?

Domain	Judgement						
Desirable effects	Trivial	Small	Moderate	Large		Varies	**Don't know**
Undesirable effects	Large	Moderate	Small	Trivial		Varies	**Don't know**
Certainty of evidence	Very low	Low	Moderate	High			**No included studies**
Values	**Important uncertainty or variability**	Possibly important uncertainty or variability	Probably no important uncertainty or variability	No important uncertainty or variability			
Balance of effects	Favours the comparison	Probably favours the comparison	Does not favour either the intervention or the comparison	Probably favours the intervention	Favours the intervention	Varies	**Don't know**

Table (Continued)

Domain	Judgement						
Resources required	Large costs	Moderate costs	Negligible costs and savings	Moderate savings	Large savings	Varies	Don't know
Cost-effectiveness	Favours the comparison	Probably favours the comparison	Does not favour either the intervention or the comparison	Probably favours the intervention	Favours the intervention	Varies	**No included studies**
Equity	Reduced	Probably reduced	Probably no impact	Probably increased	Increased	**Varies**	Don't know
Acceptability	No	Probably no	Probably yes	Yes		Varies	Don't know
Feasibility	No	Probably no	Probably yes	Yes		Varies	Don't know

PICO 12: In patients with acute central nervous system (CNS) injury [Population], is the use of a restrictive transfusion threshold [Intervention] effective to reduce mortality and improve other clinical outcomes [Outcome] compared to a liberal transfusion threshold [Comparison]?

Domain	Judgement						
Desirable effects	Trivial	**Small**	Moderate	Large		Varies	Don't know
Undesirable effects	Large	Moderate	Small	**Trivial**		Varies	Don't know
Certainty of evidence	**Very low**	Low	Moderate	High			No included studies
Values	Important uncertainty or variability	**Possibly important uncertainty or variability**	Probably no important uncertainty or variability	No important uncertainty or variability			
Balance of effects	Favours the comparison	Probably favours the comparison	**Does not favour either the intervention or the comparison**	Probably favours the intervention	Favours the intervention	Varies	Don't know
Resources required	Large costs	Moderate costs	Negligible costs and savings	Moderate savings	Large savings	Varies	Don't know
Cost-effectiveness	Favours the comparison	Probably favours the comparison	Does not favour either the intervention or the comparison	Probably favours the intervention	Favours the intervention	Varies	**No included studies**
Equity	Reduced	Probably reduced	Probably no impact	Probably increased	Increased	Varies	Don't know
Acceptability	No	Probably no	Probably yes	Yes		Varies	Don't know
Feasibility	No	Probably no	Probably yes	Yes		Varies	Don't know

PICO 13: In patients with cerebral perfusion disorders [Population], is the use of a restrictive transfusion threshold [Intervention] effective to reduce mortality and improve other clinical outcomes [Outcome] compared to a liberal transfusion threshold [Comparison]?

Domain	Judgement						
Desirable effects	Trivial	Small	Moderate	Large		Varies	**Don't know**
Undesirable effects	Large	Moderate	Small	Trivial		Varies	**Don't know**
Certainty of evidence	Very low	Low	Moderate	High			**No included studies**
Values	Important uncertainty or variability	Possibly important uncertainty or variability	Probably no important uncertainty or variability	No important uncertainty or variability			
Balance of effects	Favours the comparison	Probably favours the comparison	Does not favour either the intervention or the comparison	Probably favosurs the intervention	Favours the intervention	Varies	**Don't know**
Resources required	Large costs	Moderate costs	Negligible costs and savings	Moderate savings	Large savings	Varies	Don't know
Cost-effectiveness	Favours the comparison	Probably favours the comparison	Does not favour either the intervention or the comparison	Probably favours the intervention	Favours the intervention	Varies	No included studies
Equity	Reduced	Probably reduced	Probably no impact	Probably increased	Increased	Varies	Don't know

Table (Continued)

Domain	Judgement						
Acceptability	No	Probably no	Probably yes	Yes		Varies	Don't know
Feasibility	No	Probably no	Probably yes	Yes		Varies	Don't know

PICO 14: In patients with acute bleeding [Population], is the use of a restrictive transfusion threshold [Intervention] effective to reduce mortality and improve other clinical outcomes [Outcome] compared to a liberal transfusion threshold [Comparison]?

Domain	Judgement						
Desirable effects	Trivial	Small	Moderate	Large		Varies	**Don't know**
Undesirable effects	Large	Moderate	Small	Trivial		Varies	**Don't know**
Certainty of evidence	Very low	Low	Moderate	High			**No included studies**
Values	Important uncertainty or variability	Possibly important uncertainty or variability	Probably no important uncertainty or variability	No important uncertainty or variability			
Balance of effects	Favours the comparison	Probably favours the comparison	Does not favour either the intervention or the comparison	Probably favours the intervention	Favours the intervention	Varies	**Don't know**
Resources required	Large costs	Moderate costs	Negligible costs and savings	Moderate savings	Large savings	Varies	Don't know
Cost-effectiveness	Favours the comparison	Probably favours the comparison	Does not favour either the intervention or the comparison	Probably favours the intervention	Favours the intervention	Varies	No included studies
Equity	Reduced	Probably reduced	Probably no impact	Probably increased	Increased	Varies	Don't know
Acceptability	No	Probably no	Probably yes	Yes		Varies	Don't know
Feasibility	No	Probably no	Probably yes	Yes		Varies	Don't know

PICO 15: Is a PBM programme [Intervention] effective to improve clinical and economic outcomes [Outcome] compared to no PBM programme [Comparison]?

Domain	Judgement						
Desirable effects	Trivial	Small	**Moderate**	Large		Varies	Don't know
Undesirable effects	Large	Moderate	Small	Trivial		Varies	**Don't know**
Certainty of evidence	Very low	**Low**	Moderate	High			No included studies
Values	Important uncertainty or variability	Possibly important uncertainty or variability	**Probably no important uncertainty or variability**	No important uncertainty or variability			
Balance of effects	Favours the comparison	Probably favours the comparison	Does not favour either the intervention or the comparison	**Probably favours the intervention**	Favours the intervention	Varies	Don't know
Resources required	Large costs	Moderate costs	Negligible costs and savings	Moderate savings	Large savings	**Varies**	Don't know
Cost-effectiveness	Favours the comparison	Probably favours the comparison	Does not favour either the intervention or the comparison	Probably favours the intervention	Favours the intervention	Varies	**No included studies**

Table (Continued)

Domain	Judgement						
Equity	Reduced	Probably reduced	Probably no impact	**Probably increased**	Increased	Varies	Don't know
Acceptability	No	Probably no	**Probably yes**	Yes		Varies	Don't know
Feasibility	No	Probably no	**Probably yes**	Yes		Varies	Don't know

PICO 16: Is a specific behavioural intervention to promote the implementation of a PBM programme [Intervention] more effective to improve clinical and economic outcomes [Outcome] compared to no/another behavioural intervention[Comparison]?

Domain	Judgement						
Desirable effects	Trivial	Small	**Moderate**	Large		Varies	Don't know
Undesirable effects	Large	Moderate	Small	Trivial		Varies	**Don't know**
Certainty of evidence	**Very low**	Low	Moderate	High			No included studies
Values	Important uncertainty or variability	Possibly important uncertainty or variability	**Probably no important uncertainty or variability**	No important uncertainty or variability			
Balance of effects	Favours the comparison	Probably favours the comparison	Does not favour either the intervention or the comparison	**Probably favours the intervention**	Favours the intervention	Varies	Don't know
Resources required	Large costs	Moderate costs	Negligible costs and savings	Moderate savings	Large savings	**Varies**	Don't know
Cost-effectiveness	Favours the comparison	Probably favours the comparison	Does not favour either the intervention or the comparison	Probably favours the intervention	Favours the intervention	Varies	**No included studies**
Equity	Reduced	Probably reduced	Probably no impact	**Probably increased**	Increased	Varies	Don't know
Acceptability	No	Probably no	Probably yes	**Yes**		Varies	Don't know
Feasibility	No	Probably no	**Probably yes**	Yes		Varies	Don't know

PICO 17: Is a specific decision support system to promote the implementation of a PBM programme [Intervention] more effective to improve clinical and economic outcomes [Outcome] compared to no intervention or another decision support system/behavioural intervention [Comparison]?

Domain	Judgement						
Desirable effects	Trivial	Small	**Moderate**	Large		Varies	Don't know
Undesirable effects	Large	Moderate	Small	**Trivial**		Varies	Don't know
Certainty of evidence	Very low	**Low**	Moderate	High			No included studies
Values	Important uncertainty or variability	Possibly important uncertainty or variability	**Probably no important uncertainty or variability**	No important uncertainty or variability			
Balance of effects	Favours the comparison	Probably favours the comparison	Does not favour either the intervention or the comparison	**Probably favours the intervention**	Favours the intervention	Varies	Don't know
Resources required	Large costs	Moderate costs	Negligible costs and savings	Moderate savings	Large savings	**Varies**	Don't know
Cost-effectiveness	Favours the comparison	Probably favours the comparison	Does not favour either the intervention or the comparison	Probably favours the intervention	Favours the intervention	Varies	**No included studies**
Equity	Reduced	Probably reduced	Probably no impact	**Probably increased**	Increased	Varies	Don't know
Acceptability	No	Probably no	**Probably yes**	Yes		Varies	Don't know
Feasibility	No	Probably no	Probably yes	Yes		**Varies**	Don't know

NOTES

Bold values represent the final judgements made by the decision-making panel.

Appendix 8

Draft recommendations of the decision-making panels at the end of day 1

Definition and diagnosis of preoperative anaemia (PICO 1–2)

- The panel recognizes that perioperative anaemia is an important risk factor for perioperative morbidity and mortality and therefore recommends to detect and classify anaemia early before major elective surgery (strong recommendation based on low certainty in the evidence of effects).
- The panel noticed that the thresholds for definition of anaemia are heterogeneous in the literature. Therefore, exact thresholds need to be addressed in future studies.

Treatment of preoperative anaemia (PICO 3)

- The panel decided not to recommend the use of prophylactic transfusion in adult perioperative elective surgery patients because there is no evidence of any advantage for this approach.
- The panel recommends using iron supplementation in adult preoperative elective surgery patients with iron deficiency anaemia to reduce RBC transfusion rate (strong recommendation based on low certainty in the evidence of effects). The panel recognizes that the current evidence is based only on studies published until 2015.
- The panel decided to formulate a recommendation for further research to address the effect of iron substitution on the subgroup of patients with preoperative iron deficiency.
- The panel decided to formulate no recommendation on the use of ESA monotherapy in adult preoperative elective cardiac surgery patients.
- The panel suggests not to use ESA therapy routinely in anaemic adult preoperative elective surgery patients (conditional recommendation based on low certainty in the evidence of effects).
- The panel suggests to use ESA therapy in addition to iron supplementation in adult preoperative elective major orthopaedic surgery patients with Hb levels <13 g/dl as desirable effect (reduced RBC transfusion rate) may outweigh potential undesirable effects for this subgroup of patients (conditional recommendation based on low certainty in the evidence of effects).
- The panel decided to formulate a recommendation for further research on the use of ESA+iron therapy in adult preoperative elective surgery patients with focus on long-term (un)desirable effects, optimal dose, type of surgery (particular in cancer surgery), co-presence of iron deficiency and cost-effectiveness.

The use of RBC transfusion triggers (PICO 4–PICO 14)

- Critical care but clinically stable ICU (PICO 4–PICO 8): the panel drafted a strong recommendation in favour of using a RBC transfusion threshold of <7 g/dl for the treatment of anaemia in critically ill adult patients who are not actively bleeding (strong recommendation, moderate level of evidence). This recommendation may not apply to patients with a history of coronary heart disease, other cardiovascular disease or brain injury. Patients with septic shock are part of this population (originally separate PICO 8) and the Hb <7 g/dl trigger represents the value used in the included trials.
- Orthopaedic surgery (PICO 5): the panel drafted a conditional recommendation in favour of using a RBC transfusion threshold of Hb <8 g/dl in patients with hip fracture with cardiovascular disease or risk factors (conditional recommendation, moderate level of evidence). This recommendation was justified by the fact that no effect on mortality (although wide 95% confidence interval) or functional outcomes (walk independently at 60 days) was present. However, uncertainty regarding undesirable effects such as acute myocardial infarction resulted in a conditional rather than a strong recommendation. The trigger of Hb <8 g/dl represents the value used in the included trials and major evidence gaps in the areas are still present. Therefore, an additional recommendation for further research was proposed.
- Non-cardiac surgery (PICO 5): the panel drafted a recommendation for further research on the use of restrictive transfusion triggers in non-cardiac surgery populations. A conditional recommendation for either strategy cannot be made because of the concern over the possibility for undesirable effects in the restrictive group.
- Acute gastrointestinal bleeding (PICO 6): the panel suggested to formulate a recommendation in favour of the use of a RBC transfusion threshold of Hb 7–8 g/dl in patients with acute gastrointestinal bleeding who are haemodynamically stable (conditional recommendation, low level of evidence). This proposal was justified by the evidence from the two

included trials showing a lower mortality and a reduced RBC exposure and utilization in the restrictive transfusion group. It was noted by the panel that (1) the population in the PICO was defined as "acute gastrointestinal bleeding" whereas the study populations of the included studies were limited to acute "upper" gastrointestinal bleeding. (2) No trials identified patients with lower gastrointestinal bleeding. (3) Guidelines should emphasize that in the acutely bleeding patient, Hb is not the deciding factor for transfusion and (4) the included trials used Hb triggers (e.g. Hb <7 g/dl) to achieve specified Hb target ranges (e.g. Hb 7–9 g/dl).

- Coronary heart disease (PICO 7): the panel drafted a recommendation for further research on the use of restrictive transfusion triggers in adult patients with acute coronary syndrome or other ischaemic heart disease. This was justified by the overall low level of evidence and concern regarding undesirable effects on clinical outcomes (e.g. 30-day mortality) with a restrictive strategy.
- Cardiac surgery (PICO 9): the panel drafted a strong recommendation in favour of using a RBC transfusion threshold of <7·5 g/dl in adult cardiac surgery patients (strong recommendation, moderate level of evidence). The trigger Hb <7·5 g/dl represents the value used in the included trials, and no evidence of increased mortality or other undesirable effects was present together with a substantial reduction in RBC exposure and utilization.
- Haematology (PICO 10): no RBC transfusion trigger was recommended because of insufficient evidence (only two pilot studies in acute leukaemia (n = 149)) and no signal for undesirable effects. A recommendation for further research on RBC transfusion support in adult patients with haematological diseases (including non-malignant conditions such as haemoglobinopathies) was drafted.
- Oncology (PICO 11): no RBC transfusion trigger was recommended because no evidence was available. The panel decided that the only available trial was conducted in a postoperative surgical oncology setting in an intensive care unit and was therefore classified under PICO 5 (non-cardiac surgery).
- Neurology – Central nervous system injuries (PICO 12): no RBC transfusion trigger was recommended due to a very low level of evidence for all (critical) outcomes. The panel decided to formulate a recommendation for further research on the use of restrictive transfusion triggers in patients with CNS injury.
- Neurology – Cerebral perfusion disorders (PICO 13): no RBC transfusion trigger was recommended because no evidence for any outcome related to a restrictive transfusion strategy was available. Indeed, the included trial randomised patients to a Hb trigger of 10 g/dl (intervention group) or 11.5 g/dl (control group). The intervention group was not considered as a restrictive transfusion strategy group.
- Acute bleeding (PICO 14): because only evidence from one pseudo-randomized trial from 1956 was available, no RBC transfusion trigger was recommended. The panel view was that a Hb concentration alone should not be used to determine the need for transfusion in an acutely bleeding scenario (i.e. major haemorrhage)

Implementation of PBM programmes (PICO 15-17)

- The panel drafted a recommendation in favour of using comprehensive PBM programmes to improve appropriate RBC utilization (conditional recommendation based on low certainty in the evidence of effects).
- The panel drafted a recommendation in favour of using behavioural interventions (transfusion guideline/audit/form/education) to improve appropriate RBC utilization (conditional recommendation based on very low certainty in the evidence of effects).
- The panel drafted a recommendation in favour of using electronic/computerized decision support systems to improve appropriate RBC utilization (conditional recommendation based on low certainty in the evidence of effects).
- General research priorities in the field of PBM implementation were formulated including (1) measuring the impact on FFP/PLT/cryo utilization and clinical outcomes, (2) designing well-conducted observational studies (e.g. time interrupted series), (3) the assessment of compliance data, (4) measuring the cost-effectiveness of PBM programmes and the relative effectiveness of different types of decision support systems.
